# A model based on CT radiomic features for predicting RT-PCR becoming negative in coronavirus disease 2019 (COVID-19) patients

**DOI:** 10.1186/s12880-020-00521-z

**Published:** 2020-10-20

**Authors:** Quan Cai, Si-Yao Du, Si Gao, Guo-Liang Huang, Zheng Zhang, Shu Li, Xin Wang, Pei-Ling Li, Peng Lv, Gang Hou, Li-Na Zhang

**Affiliations:** 1 Department of Emergency Medicine, The First Affiliated Hospital of China Medical University, Nanjing North Street 155, Shenyang, 110001 Liaoning Province China; 2 Department of Radiology, The First Affiliated Hospital of China Medical University, Shenyang, 110001 China; 3 Department of Pulmonary and Critical Care Medicine, The First Affiliated Hospital of China Medical University, Shenyang, 110001 China

**Keywords:** COVID-19, Computed tomography, Radiomics, Quantitative, RT-PCR

## Abstract

**Background:**

Coronavirus disease 2019 (COVID-19) has emerged as a global pandemic. According to the diagnosis and treatment guidelines of China, negative reverse transcription-polymerase chain reaction (RT-PCR) is the key criterion for discharging COVID-19 patients. However, repeated RT-PCR tests lead to medical waste and prolonged hospital stays for COVID-19 patients during the recovery period. Our purpose is to assess a model based on chest computed tomography (CT) radiomic features and clinical characteristics to predict RT-PCR negativity during clinical treatment.

**Methods:**

From February 10 to March 10, 2020, 203 mild COVID-19 patients in Fangcang Shelter Hospital were retrospectively included (training: n = 141; testing: n = 62), and clinical characteristics were collected. Lung abnormalities on chest CT images were segmented with a deep learning algorithm. CT quantitative features and radiomic features were automatically extracted. Clinical characteristics and CT quantitative features were compared between RT-PCR-negative and RT-PCR-positive groups. Univariate logistic regression and Spearman correlation analyses identified the strongest features associated with RT-PCR negativity, and a multivariate logistic regression model was established. The diagnostic performance was evaluated for both cohorts.

**Results:**

The RT-PCR-negative group had a longer time interval from symptom onset to CT exams than the RT-PCR-positive group (median 23 vs. 16 days, *p* < 0.001). There was no significant difference in the other clinical characteristics or CT quantitative features. In addition to the time interval from symptom onset to CT exams, nine CT radiomic features were selected for the model. ROC curve analysis revealed AUCs of 0.811 and 0.812 for differentiating the RT-PCR-negative group, with sensitivity/specificity of 0.765/0.625 and 0.784/0.600 in the training and testing datasets, respectively.

**Conclusion:**

The model combining CT radiomic features and clinical data helped predict RT-PCR negativity during clinical treatment, indicating the proper time for RT-PCR retesting.

## Background

Coronavirus disease 2019 (COVID-19) is a major threat to the health of people worldwide. According to the diagnosis and treatment guidelines proposed by the National Health Committee of the People’s Republic of China (7th Edition) [1], negative reverse transcription-polymerase chain reaction (RT-PCR) is the key criterion for discharging COVID-19 patients. The clinical prediction of RT-PCR becoming negative is critical for the proper retesting time, preventing medical waste from repeated RT-PCR tests and unnecessary prolonged hospital stays. Doctors need an objective and accurate method for prediction of RT-PCR negativity during clinical treatment.

Chest computed tomography (CT) can intuitively demonstrate the lung lesions and its manifestations of COVID-19 pneumonia have been reported in many studies [2–4]. Chest CT exams are useful in supplementary diagnosis of RT-PCR tests [5–7], evaluating disease stages [2, 3, 8, 9] and severity [10–12]. Recently, deep learning techniques have been widely used in the detection and segmentation of COVID-19 lesions in chest CT images [13–16]. Based on a reliable segmentation method, the high-throughput and high-dimensional radiomic features on chest CT showed strong potential for predicting the true status of RT-PCR.

We hypothesized that a model incorporating CT radiomic features and clinical characteristics can predict RT-PCR becoming negative. We collected the clinical data and chest CT features of mild COVID-19 patients in Fangcang Shelter Hospital in Wuhan, Hubei, aiming to establish a predictive model for RT-PCR becoming negative during the recovery period.

## Patients and methods

The study was approved by the institutional review board of the First Affiliated Hospital of China Medical University. Informed consent was waived due to the nature of the retrospective study.

### Patients

**Fig. 1:**
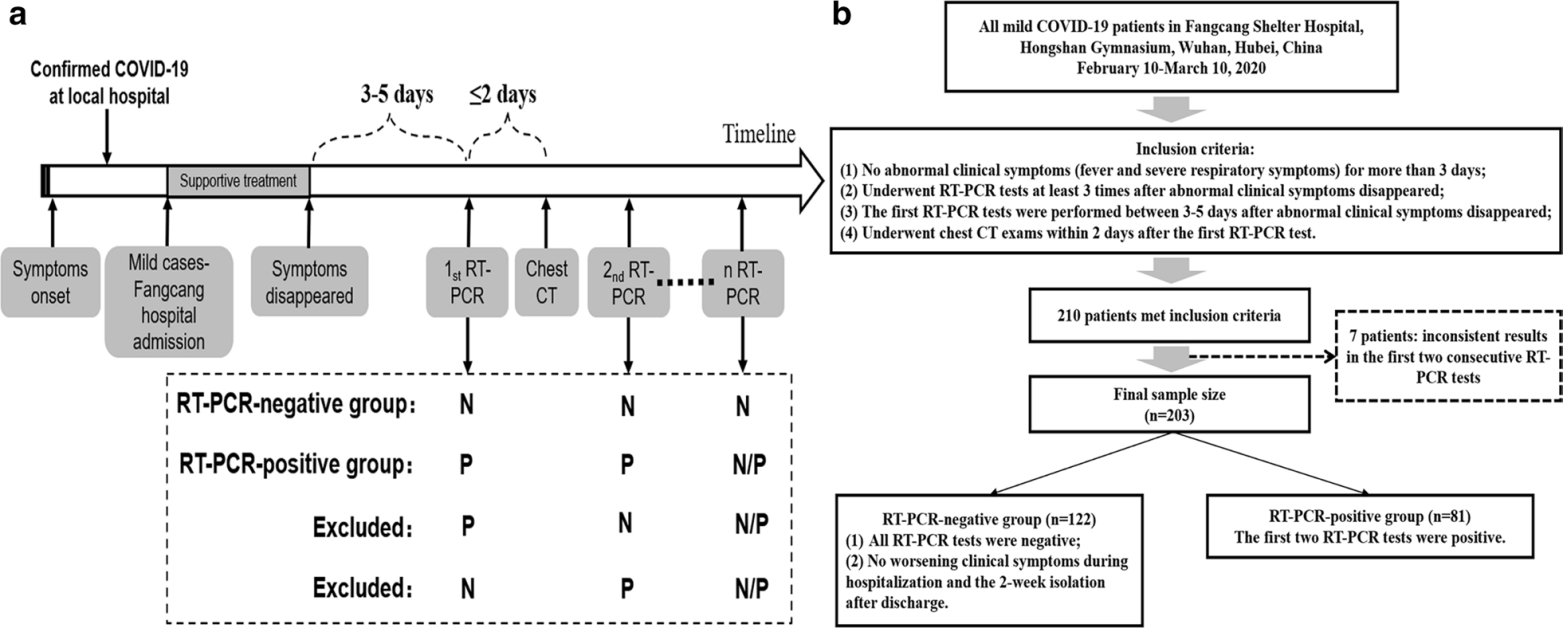
The operating mode diagram of Fangcang Shelter Hospital in our study (**a**); the flow diagram summarizing the selection of the enrolled patients (**b**). *N* negative, *P* positive

Between February 10, 2020, and March 10, 2020, the clinical data and CT images of COVID-19 patients at Fangcang Shelter Hospital in Hongshan Gymnasium, Wuhan, Hubei, were reviewed retrospectively. All cases were mild from the onset and during the course of hospitalization, as defined by no hypoxemia or respiratory distress (respiratory rate ≥ 30 breaths/min, requirement for oxygen treatment or mechanical ventilation, or SpO2 ≤ 93% on room air) [1]. Patients were included if they met the following criteria: (1) No abnormal clinical symptoms (fever and severe respiratory symptoms) for more than 3 days. (2) Underwent RT-PCR tests at least 3 times after abnormal clinical symptoms disappeared. (3) The first RT-PCR tests were performed between 3 and 5 days after abnormal clinical symptoms disappeared. (4) Underwent chest CT exams within 2 days after the first RT-PCR test. Patients with inconsistent results in the first two consecutive RT-PCR tests were excluded (Fig. 1a, b). Novel coronavirus 2019-nCoV nucleic acid detection kit (fluorescence PCR method) (Sansure Biological Technology Co., Ltd., Changsha, China, Serial Number: 20150036) was used for RT-PCR tests.

The enrolled patients were divided into two groups: RT-PCR-negative and RT-PCR-positive groups (Fig. 1a, b). Inclusion criteria for the RT-PCR-negative group were: (1) All RT-PCR tests were negative; (2) No worsening clinical symptoms during hospitalization and the 2-week isolation after discharge. Inclusion criteria for the RT-PCR-positive group: the first two RT-PCR tests were positive.

### Clinical characteristics

We collected 20 available clinical characteristics, including general characteristics (age, gender, time interval from symptoms onset to CT exams), comorbidities, vital signs on the CT scan day and laboratory tests on admission. Comorbidities included diabetes, hypertension, cardiovascular disease, chronic obstructive pulmonary disease, chronic liver disease and cancer. Vital signs on the CT scan day included heart rate, systolic blood pressure, diastolic blood pressure, respiratory rate, and blood oxygen saturation. Laboratory tests include white blood cell count, neutrophil count, lymphocyte count, platelet count, hemoglobin and neutrophil/lymphocyte ratio (NLR) (NLR = neutrophil counts/lymphocyte counts).

### CT protocol

The first RT-PCR tests for all enrolled patients were performed between 3 and 5 days after abnormal clinical symptoms disappeared. Then, all patients underwent CT exams within 2 days after the first RT-PCR test. Chest CT scanning used a mobile cabin CT (CT-NeuVz Prime, Neusoft) with a single breath-hold in the supine position. The scan parameters are as follows: tube voltage of 120 kVp, tube current of 100–200 mA, detector collimation of 64 or 128 × 0.625 mm, field of view of 350 mm × 350 mm, and matrix size of 512 × 512. Imaging data were reconstructed using a medium sharp reconstruction algorithm with a slice thickness of 5 mm and an interval of 1 mm.

### Image segmentation and feature extraction

**Fig. 2:**
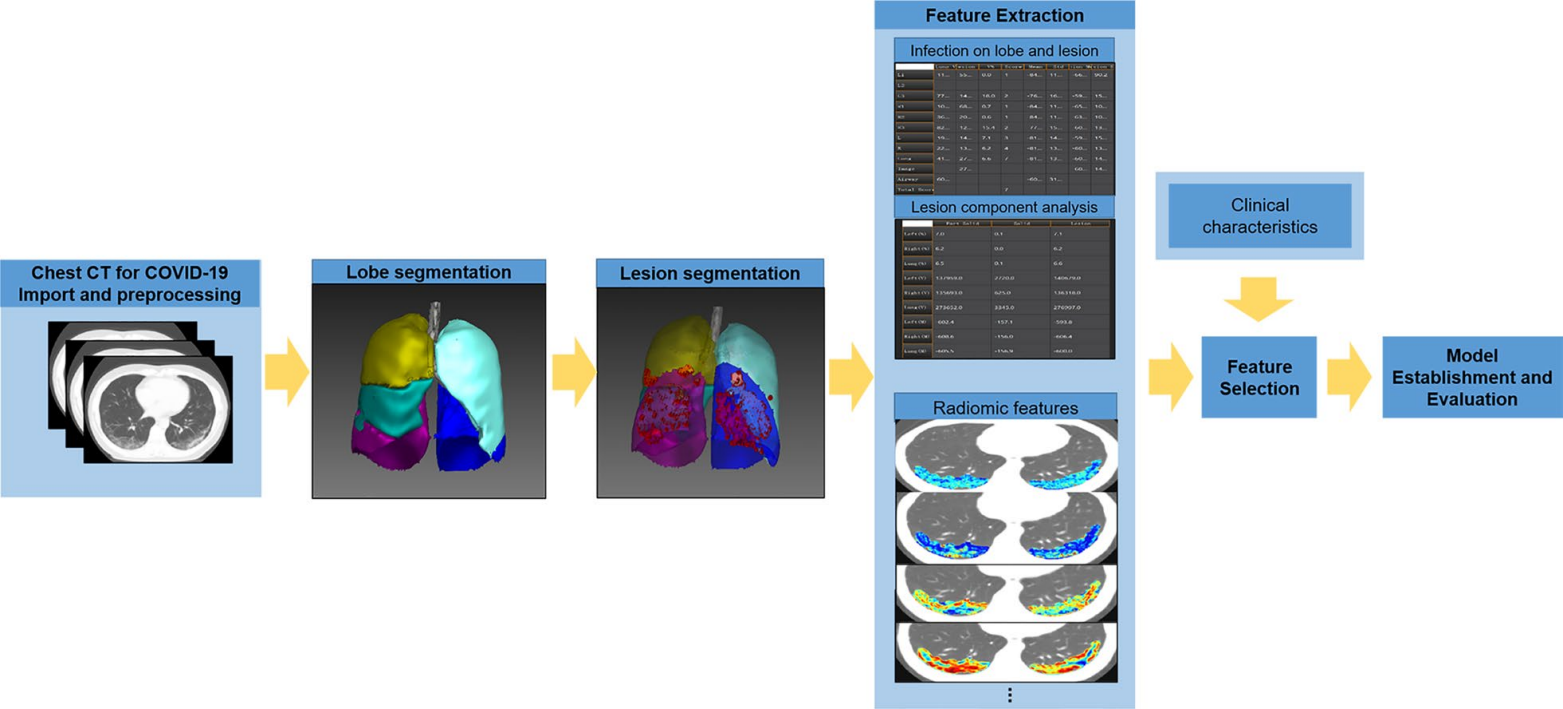
The process of model establishment

CT image analysis was performed on a dedicated workstation—Lung intelligence Kit (LK) Version V2.1.1. R (GE Healthcare, China). The main processes included data import and preprocessing, lung lobe segmentation, lesion segmentation and feature extraction (Fig. 2). Lung lobes were segmented with the purpose of improving the accuracy of lesion segmentation and calculating the proportion of lesions in each lung lobe.

#### Lobe and lesion segmentation

Before lung lobe and lesion segmentation, the images were resampled to voxel size 1 × 1 × 1 mm^3^, and a Gaussian filter was applied for denoising. Then, a fully automatic segmentation of three-dimensional lung lobes and lesions based on deep learning algorithms was performed. In cases of unsatisfactory lung lobe and lesion segmentation, two thoracic radiologists (with 5 and 15 years of experience, respectively) blinded to the clinical information and RT-PCR results manually adjusted the contour and resolved discrepancies by consensus.

#### Quantitative feature extraction

After segmentation, 86 CT quantitative parameters were automatically calculated: the statistical results of lung lobe and lesion (volume, volume percentage, pneumonia score, average density, standard deviation of density) and the component analysis of the lesion (partial solidity, solidity and total lesions) (Additional file 1: Supplementary Data 1).

#### Radiomic feature extraction

After segmentation, 120 radiomic features of 7 categories were automatically calculated: (1) first-order features (n = 19); (2) 2D and 3D shape features (n = 26); (3) gray level cooccurrence matrix features (n = 24); (4) gray level run length matrix features (n = 16); (5) gray level size zone matrix features (n = 16); (6) neighboring gray tone difference matrix features (n = 5); and (7) gray level dependence matrix features (n = 14). Detailed names and definitions of all 120 features can be found in Additional file 1: Supplementary Data 2.

### Feature selection

Missing values were replaced by the median, and the data were standardized by the following formula: standardized value = (original value-average value)/standard deviation.

The patients were randomly assigned at a 7:3 ratio to either the training cohort or the testing cohort. All patients in the training cohort were used to build the predictive model, while patients in the testing cohort were used to independently evaluate the model’s performance. To obtain the strongest features that were significantly associated with negative RT-PCR results in the training cohort, we performed univariate logistic regression analysis, and features with a *p* value < 0.10 were used for subsequent analysis. Then, Spearman correlation analysis was used to remove the features highly correlated with others; here, the |r| value was 0.9.

### Model establishment and evaluation

We constructed a multivariate logistic regression model to identify a strategy to best classify RT-PCR-negative patients in the training dataset. Radiomics scores (Rad-scores) were calculated in each patient through a linear combination of the extracted features with their respective coefficients. The predictive performance was evaluated in terms of discrimination-receiver operating characteristic (ROC) curve, calibration-calibration curve and clinical application-decision curve.

### Statistical analysis

Categorical variables are presented as the number and percentage of the total. The normality of continuous variables was evaluated by using the Shapiro–Wilk test. Normally distributed variables are shown as the mean ± standard deviation or the median (25% percentile, 75% percentile). The differences in variables between different subgroups were assessed by the t test or Mann–Whitney U test as appropriate. The chi-squared test was used to compare the significance of the differences between categorical variables. All statistical analyses for the present study were performed with R 3.5.1 and Python 3.5.6. A two-tailed *p* value < 0.05 indicated statistical significance.

## Results

### Analysis of clinical and CT quantitative features

**Fig. 3:**
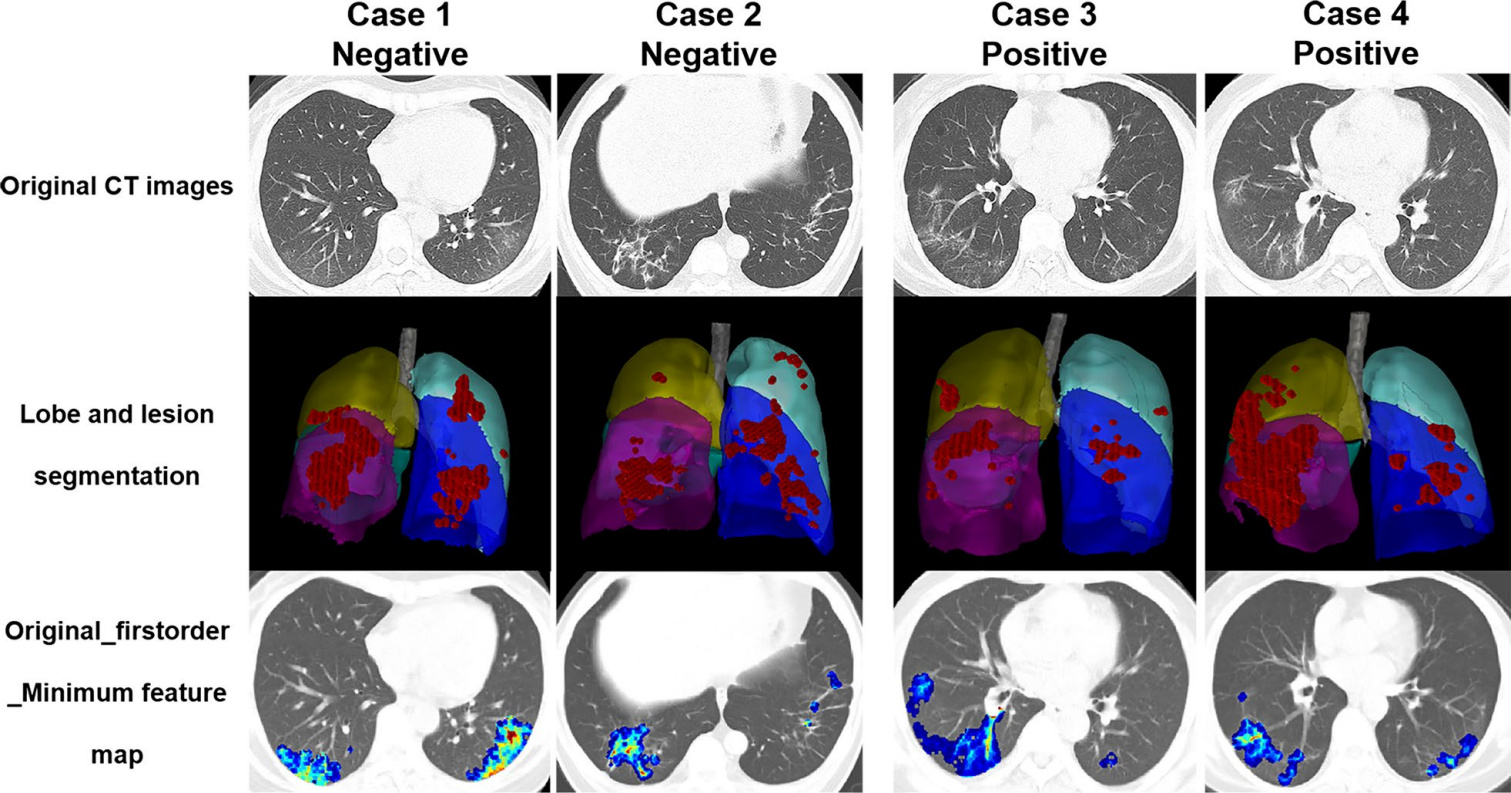
CT images for cases in the RT-PCR-negative and RT-PCR-positive groups. The CT exams were performed on the 31st, 23rd, 24th and 22nd days from symptom onset for case 1–4, respectively. The two groups have a great overlap in original CT images and are difficult to be distinguished by eyes. After deep learning-based lobe and lesion segmentation, the radiomic feature maps of the lesion are calculated. For example, we can observe that original_firstorder_Minimum of the RT-PCR-negative group seems higher than the RT-PCR-positive group

The flow diagram summarizing the selection of the enrolled patients is shown in Fig. 1b. For 203 patients included in our study, the average number of RT-PCR tests was 6 ± 3, ranging from 3 to 12 during hospitalization. 122/203 (60.1%) were categorized in the RT-PCR-negative group, and 81 (39.9%) were categorized in the RT-PCR-positive group. Figure 3 shows CT images for cases in the RT-PCR-negative and RT-PCR-positive groups. Clinical information of the training and the testing cohort is shown in Table 1. The RT-PCR-negative group had a longer time interval from symptom onset to CT exams than the RT-PCR-positive group (median 23 vs. 16 days for the total patients, *p* < 0.001). There was no significant difference in the other clinical characteristics. The CT quantitative features are summarized in Additional file 1: Supplementary Data 1, and none of them differed between the two groups.

**Table 1:** Clinical characteristics of the RT-PCR-negative and RT-PCR-positive groups

Variables	Total (n = 203)			Training dataset (n = 141)			Testing dataset (n = 62)		
	Negative (n = 122)	Positive (n = 81)	*p*	Negative (n = 85)	Positive (n = 56)	*p*	Negative (n = 37)	Positive (n = 25)	*p*
General characteristics									
Age (years)	52 (41, 58)	49 (39, 57)	0.325	52 (41, 61)	51 (39, 58)	0.551	52 (41, 56)	46 (38, 56)	0.315
Gender			0.343			0.191			0.800
Female, n (%)	70 (57.38)	41 (50.62)		49 (57.65)	26 (46.43)		21 (56.76)	15 (60.00)	
Male, n (%)	52 (42.62)	40 (49.38)		36 (42.35)	30 (53.57)		16 (43.24)	10 (40.00)	
Time interval from symptoms onset to CT exams (days)	23 (18, 30)	16 (10, 22)	< 0.001	22 (18, 30)	17 (11, 22)	< 0.001	25.16 ± 8.45	15.52 ± 7.51	**< 0.001**
Comorbidities									
Diabetes, n (%)	9 (7.38)	1 (1.23)	0.099	7 (8.24)	1 (1.79)	0.212	2 (5.41)	0 (0.00)	0.511
Hypertension, n (%)	20 (16.39)	10 (12.35)	0.426	13 (15.29)	9 (16.07)	0.901	7 (18.92)	1 (4.00)	0.183
Cardiovascular disease, n (%)	1 (0.82)	1 (1.23)	1.000	1 (1.18)	1 (1.79)	1.000	0 (0.00)	0 (0.00)	1.000
COPD, n (%)	4 (3.28)	2 (2.47)	0.929	2 (2.35)	1 (1.79)	0.713	2 (5.41)	1 (4.00)	0.726
Chronic liver disease, n (%)	1 (0.82)	0 (0.00)	1.000	1 (1.18)	0 (0.00)	1.000	0 (0.00)	0 (0.00)	1.000
Cancers, n (%)	3 (2.46)	1 (1.23)	0.921	1 (1.18)	1 (1.79)	1.000	2 (5.41)	0 (0.00)	0.511
Vital signs*									
Heart rate (beats/minute)	83 (76, 90)	85 (76, 90)	0.650	83 (74, 90)	85 (78, 89)	0.432	84.51 ± 10.90	83.40 ± 14.06	0.727
Systolic blood pressure (mmHg)	127 (121, 135)	128 (120, 134)	0.637	126 (121, 133)	129 (121, 135)	0.822	129.43 ± 11.90	126.40 ± 12.53	0.339
Diastolic blood pressure (mmHg)	78 (74, 84)	79 (74, 84)	0.621	78 (73, 84)	79 (72, 85)	0.420	79 (75, 87)	80 (76, 83)	0.841
Respiratory rate (times/minute)	19 (18, 20)	19 (18, 20)	0.430	19 (18, 20)	19 (18, 20)	0.703	19 (18, 20)	19 (19, 20)	0.393
Blood oxygen saturation (%)	96 (96, 97)	96 (96, 98)	0.887	96 (96, 97)	96 (96, 98)	0.654	97 (96, 98)	96 (96, 97)	0.621
Laboratory indicators*									
White blood cell count (× 10^9^/L)	5.19 (4.62, 6.27)	5.10 (4.18, 5.86)	0.194	5.16 (4.53, 6.27)	5.00 (4.11, 5.61)	0.298	5.40 (4.75, 6.23)	5.19 (4.40, 6.18)	0.478
Neutrophil count (× 10^9^/L)	3.13 (2.66, 3.75)	2.79 (2.34, 3.46)	0.061	3.14 (2.46, 3.73)	2.83 (2.33, 3.46)	0.166	3.11 (2.72, 3.87)	2.79 (2.31, 3.49)	0.192
Lymphocyte count (× 10^9^/L)	1.64 (1.33, 1.97)	1.66 (1.30, 2.01)	0.964	1.58 (1.33, 1.91)	1.60 (1.26, 2.00)	0.982	1.76 (1.43, 2.02)	1.78 (1.43, 2.06)	0.914
Hemoglobin count (g/L)	127 (118, 140)	129 (118, 139)	0.576	126.89 ± 16.99	131.46 ± 15.33	0.107	131 (121, 141)	122 (115, 133)	0.092
Platelet count (× 10^9^/L)	232 (180, 279)	219 (183, 283)	0.469	226 (177, 281)	219 (172, 263)	0.565	240 (187, 277)	215 (191, 288)	0.651
NLR	1.83 (1.49, 2.40)	1.77 (1.34, 2.16)	0.160	1.91 (1.43, 2.50)	1.79 (1.36, 2.25)	0.357	1.82 (1.57, 2.13)	1.62 (1.24, 2.02)	0.187

Bold with *p* value < 0.05. Categorical variables are presented as numbers (percentages). Quantitative variables are presented as the mean ± standard deviation or median (25% percentile, 75% percentile) according to normality test results

*COPD* chronic obstructive pulmonary disease, *NLR* neutrophil/lymphocyte ratio

*Normal range: heart rate, 60–100 beats/minute; Systolic blood pressure, 90–140 mmHg; Diastolic blood pressure, 60–90 mmHg; Respiratory rate, 12–20 times/minute; Blood oxygen saturation, 95–100%; White blood cell count, 3.50–9.50 × 10^9^/L; Neutrophil count, 1.80–6.30 × 10^9^/L; Lymphocyte count, 1.10–3.20 × 10^9^/L; Hemoglobin count, 130–175 g/L; Platelet count, 125–350 × 10^9^/L

### Feature selection

A total of 226 characteristics from each patient were collected: 20 clinical characteristics, 86 quantitative features and 120 radiomic features. After the univariate logistic regression analysis was performed, 20/226 parameters were reserved. Then, 10 features that were highly correlated (|r|> 0.9) with other features were removed due to their redundancy based on the Spearman correlation analysis. Ultimately, 10/20 parameters (Table 2) were retained to build the model.

**Table 2:** Statistical summary of the multivariate logistic regression model

Variables	Coefficient	Std. error	Wald	OR (95% CI)
Time interval from symptoms onset to CT exams	1.045	0.257	16.587	2.84 (1.72, 4.70)
Original_firstorder_Minimum	0.740	0.619	1.430	2.10 (0.62, 7.04)
Original_gldm_SmallDependenceLowGrayLevelEmphasis	0.187	0.297	0.397	1.21 (0.67, 2.16)
Original_glszm_LargeAreaHighGrayLevelEmphasis	0.093	0.290	0.104	1.10 (0.62, 1.94)
Original_firstorder_10Percentile	0.050	0.616	0.007	1.05 (0.31, 3.52)
Original_shape_Sphericity	− 0.007	0.279	0.001	0.99 (0.57, 1.72)
Original_gldm_LargeDependenceLowGrayLevelEmphasis	− 0.076	0.302	0.063	0.93 (0.51, 1.67)
Original_gldm_LargeDependenceHighGrayLevelEmphasis	− 0.123	0.350	0.122	0.88 (0.45, 1.76)
Original_glrlm_ShortRunHighGrayLevelEmphasis	− 0.183	0.328	0.306	0.83 (0.44, 1.59)
Original_shape_SurfaceArea	− 0.570	0.309	3.402	0.57 (0.31, 1.04)
Constant	0.609			

*OR* odds ratio, *CI* confidence interval

### Model establishment and evaluation

**Fig. 4:**
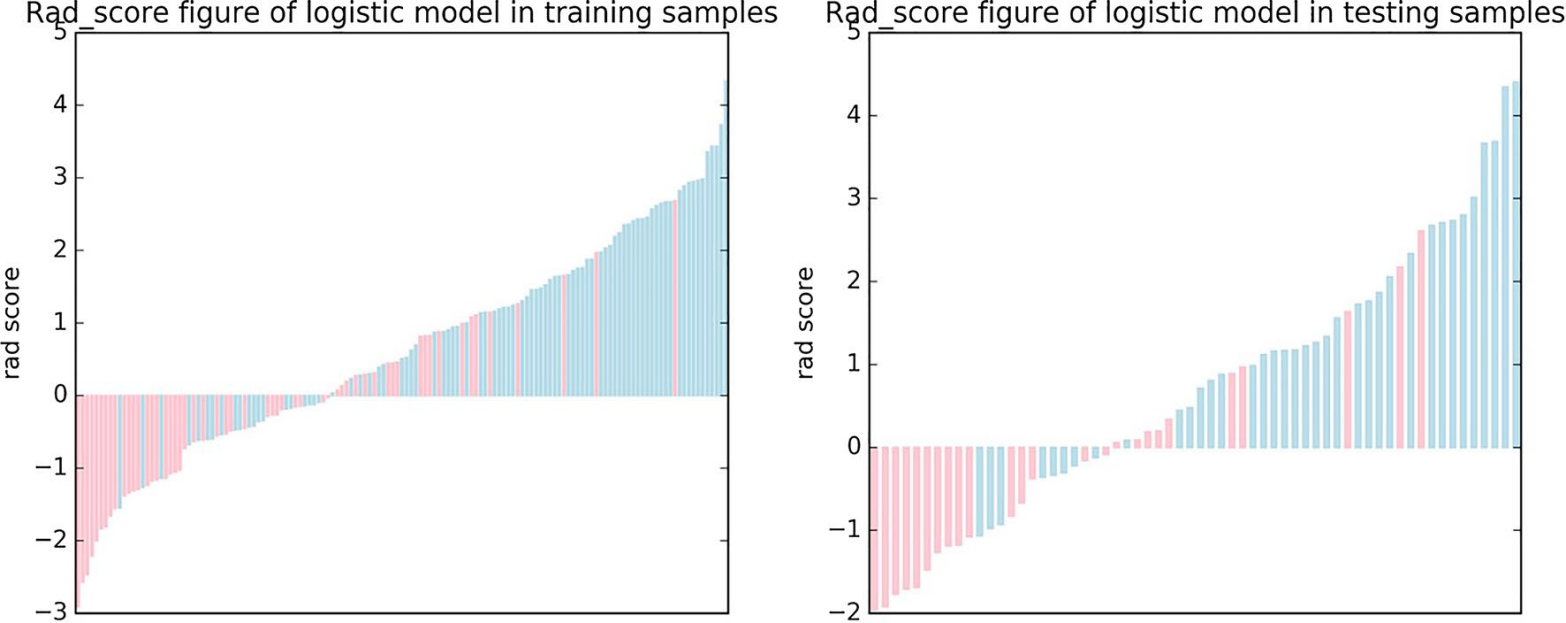
Rad scores for each patient in the training and testing cohorts

**Fig. 5:**
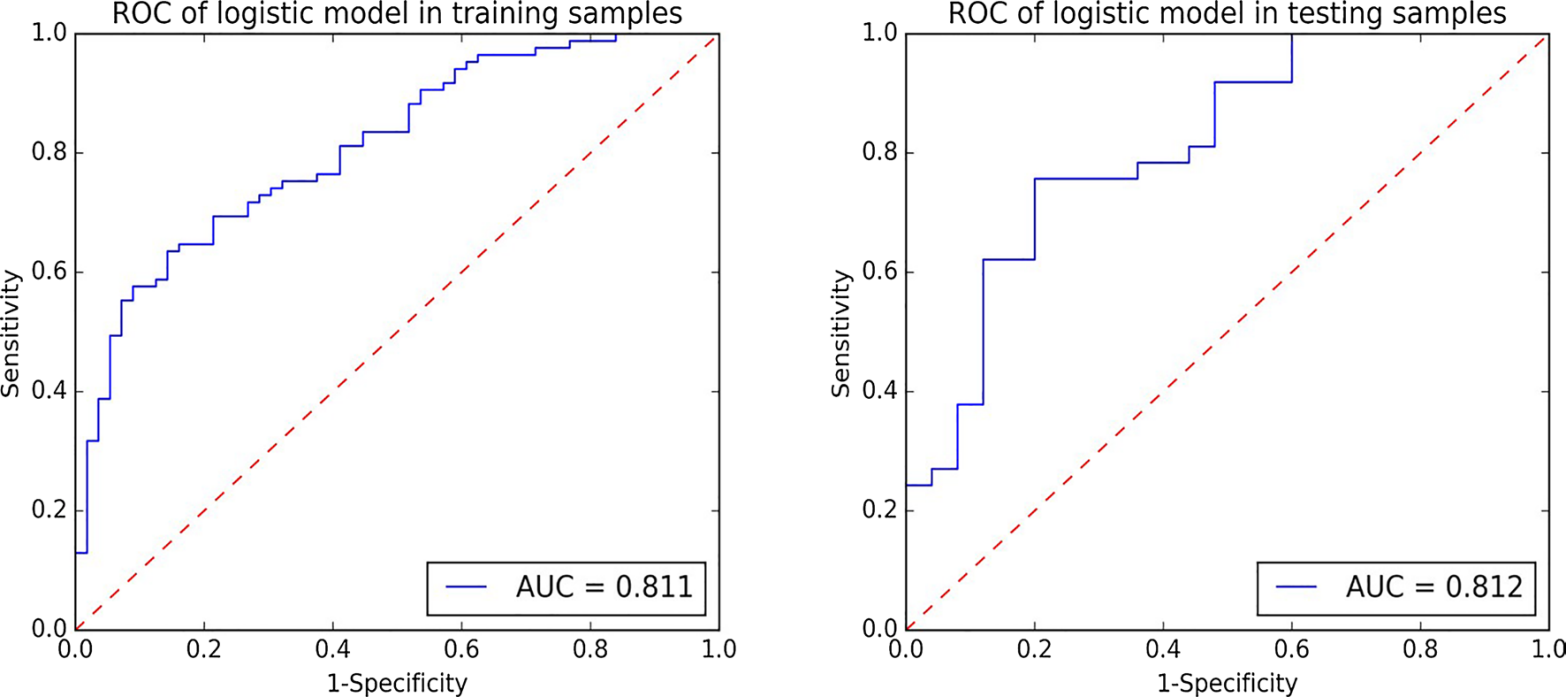
ROC curve in the training and testing datasets. *ROC* receiver operating characteristic

**Fig. 6:**
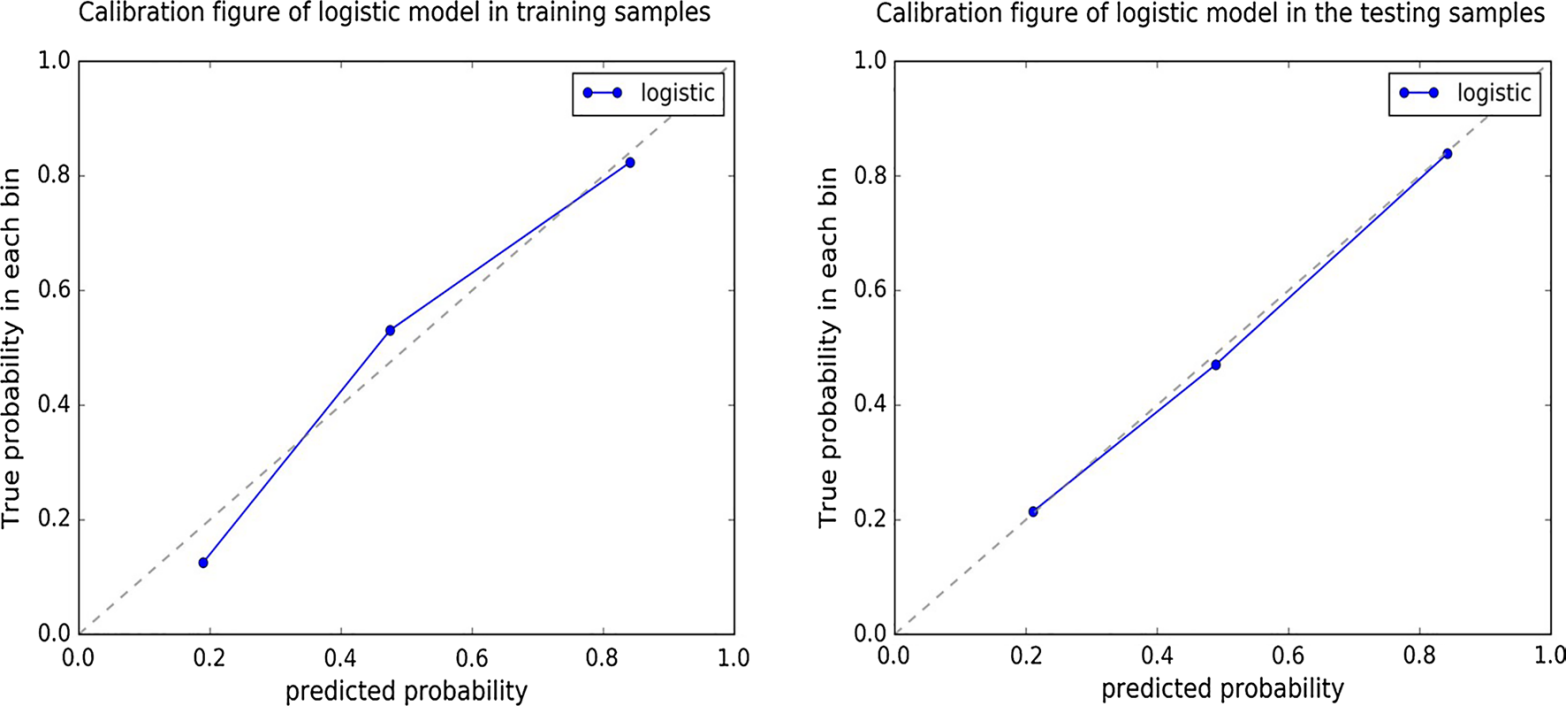
Calibration curve in the training and testing datasets. The y-axis shows the actual result. The x-axis represents the predicted probability. The diagonal dotted line represents an ideal model. The blue solid line indicates the performance of the model. The closer the blue solid line is to the diagonal dotted line, the better the prediction is

**Fig. 7:**
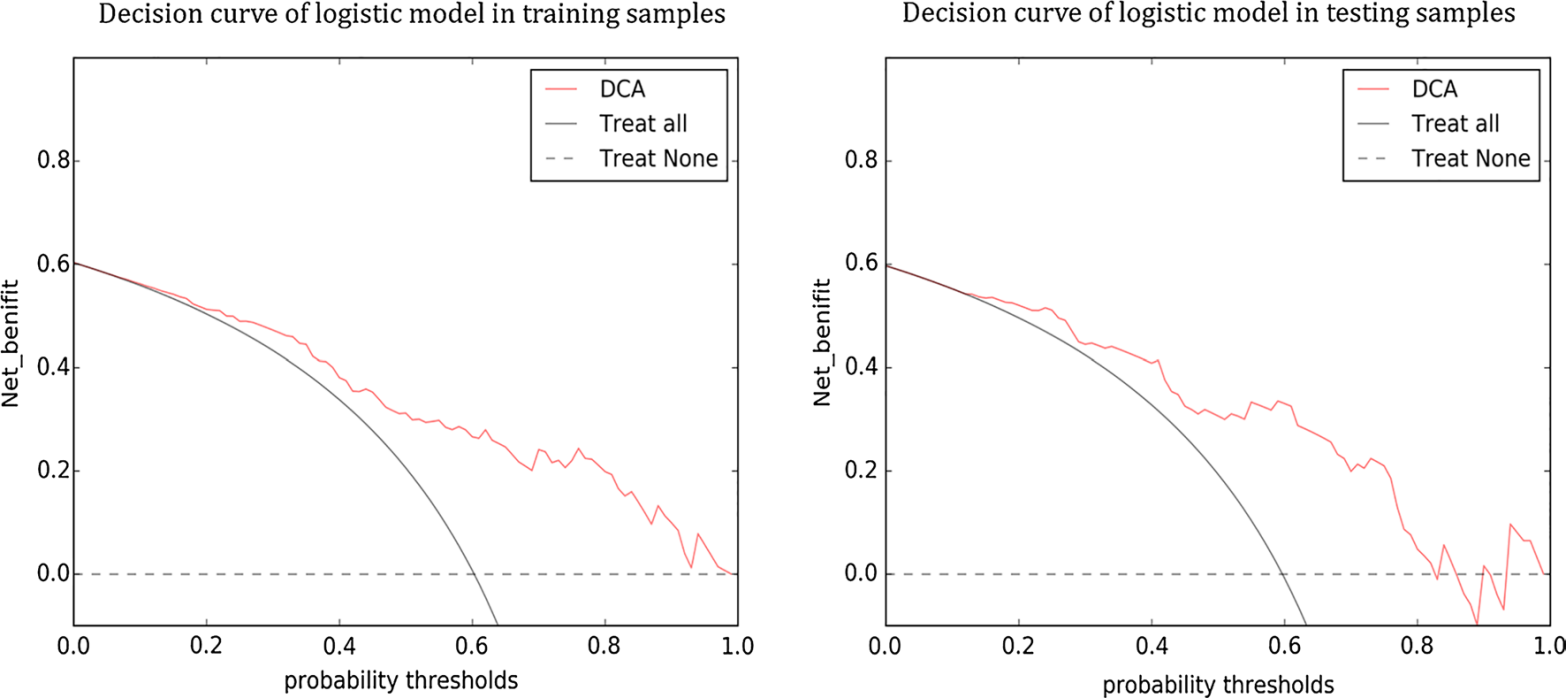
Decision curve analysis (DCA) in the training and testing datasets. The y-axis represents the net benefit (the net benefit was calculated by subtracting the proportion of all false-positive patients from the true-positive patient, and the weight is the relative hazard of abandoning treatment versus negative patients). The red solid line indicates the model. The black solid line indicates the hypothesis that all patients were treated by one scheme (for example, assuming that all patients were in the RT-PCR-negative group). The black dotted line represents the hypothesis that all patients were treated by another scheme (for example, assuming that all patients were in the RT-PCR positive group). The model shows the added net benefit if the probability thresholds in the training and testing datasets are more than 0.20 and between 0.15 and 0.82, respectively

The statistical summary of the multivariate logistic regression model is shown in Table 2. The time interval from symptom onset to CT exams and original_firstorder_Minimun had the highest odds ratio (OR) values (OR = 2.84 and 2.10, respectively) among all parameters. Figure 4 shows Rad-score for each patient in the training and testing datasets. ROC curves of the model (Fig. 5) showed an area under the curve (AUC) of 0.811 with a sensitivity of 76.5%, specificity of 62.5% and accuracy of 70.9% in the training dataset and 0.812 with a sensitivity of 78.4%, specificity of 60.0% and accuracy of 71.0% in the testing dataset. The calibration curve of Rad-scores for the differentiation of the RT-PCR-negative group demonstrated the good consistency between prediction and observation in the training and testing cohorts (Fig. 6). The decision curve analysis showed that the model had a significantly improved performance within a certain threshold range in the training and testing datasets (Fig. 7).

## Discussion

We demonstrated the usefulness of CT radiomic features for predicting RT-PCR negativity and established a predictive model based on CT radiomic features combined with clinical data in COVID-19 patients during the recovery period. With AUCs of 0.811 and 0.812 for the training and testing datasets, respectively, we expect the model to help doctors effectively predict RT-PCR negativity during clinical treatment.

The unsatisfactory sensitivity of RT-PCR detection is a major concern [5, 6, 17, 18]. To avoid the possibility of false negative RT-PCR in our study, we included patients with repeated RT-PCR tests (average times: 6; range 3–12) during hospitalization. Only the patients with consistent results of the consecutive RT-PCR tests were included to ensure true negative or positive RT-PCR status for the corresponding CT. A 2-week isolation after discharge was further performed to avoid any possibility of false negative RT-PCR.

Accurate lesion segmentation is the key to feature extraction and model construction. Colombi et al.’s study [19] divided lung parenchyma into upper, middle and lower zones in severe COVID-19 patients. They found quantification of well aerated lung parenchyma were predictors of adverse outcome. In the present study, we used the automatic pneumonia segmentation software based on a deep learning algorithm. It detected the respiratory tract and lung lesions based on the actual segmentation of the lung lobes, so more comprehensive and complicated quantitative parameters and radiomic features were evaluated for model construction. Recently, the deep learning algorithm has been widely used in the detection of COVID-19 lesions in chest CT images [13–16]. Most studies [13–15] applied it to chest CT images in the early stage of the disease course for diagnosis and differential diagnosis, while there are few studies regarding chest CT images of COVID-19 patients during the recovery period. We analyzed chest CT images after the abnormal clinical symptoms disappeared, and proposed a combination model of radiomic features and clinical data to predict RT-PCR negativity.

Radiomic features played important roles in the model. Among the 10 parameters in the model, 9 of them were CT radiomic features. The top five radiomic features are original_firstorder_Minimum, original_gldm_Small Dependence Low Gray Level Emphasis, original_glszm_Large Area High Gray Level Emphasis, original_firstorder_10Percentile, and original_shape_Sphericity (Table 2). These indicators represent lesion internal heterogeneity of morphology, density, texture and distribution, thus indicating disease severity. The time interval from symptom onset was the only clinical parameter selected in the model, with the strongest correlation with the RT-PCR-negative group (OR = 2.84). As expected, the longer the disease course, the more patients received negative RT-PCR.

We also analyzed the chest CT quantitative parameters, but none of them were included in the model. Increased numbers, extents, and densities of ground-glass opacities (GGOs) [20] and consolidations [21] represent progression in COVID-19 patients, as well as the transformation of consolidation from GGOs [8]. Decreased sizes, extents, and degrees of such lesions could indicate improvement [21–25]. In our study, the recovering patients who had a negative RT-PCR result were expected to show smaller lesion volumes and lower CT values, but the quantitative parameters were not precise enough for the changes. The high-throughput and high-dimensional radiomic features could reflect more detailed changes inside the lesions than the CT quantitative parameters.

No laboratory tests were included in the model. Neutrophils and lymphocytes are the main hematological indicators reflecting systematic inflammation. Lymphocytopenia occurred in more than 80% of critically ill patients [26], while in an almost mild study population, only 35% of patients had mild lymphocytopenia [27]. Elevated baseline neutrophils in mild cases were not common, and only 6.3% of non-severe patients showed increases in Zhang et al.’s study [28]. Neutrophils also did not increase over the disease course for patients with mild disease and survivors [22, 29]. The patients included in our study were mild COVID-19 patients from Fangcang Shelter Hospital. Most laboratory tests were normal or slightly exceeded normal limits, and we did not find a significant difference in lymphocytes and neutrophils between the RT-PCR-negative and RT-PCR-positive groups.

This study has several limitations. First, as a retrospective study, the study only involved mild COVID-19 cases, so the model cannot be employed for severe and critical cases. For all mild COVID-19 patients in Fangcang Shelter Hospital, some laboratory tests such as erythrocyte sedimentation rate and C-reactive protein were not performed. Second, this is a single-center study, and multi-center data should be used for further verification. Moreover, we only built one model type and lacked comparative analysis with other model types, including decision trees, random forests and support vector machines. Finally, we did not explain the biological interpretation of the radiomic features. We are fully aware of the need for further exploration of these conclusions in subsequent studies.

## Conclusion

In conclusion, the established model based on CT radiomic features and clinical data could help doctors predict RT-PCR negativity during the clinical treatment, indicating the proper time for RT-PCR retesting.

## Supplementary information

**Additional file 1.** Names and definitions of radiomic features.

## Data Availability

The datasets generated and analyzed during the current study are available from the corresponding author on reasonable request.

## Abbreviations

COVID-19: Coronavirus disease 2019
CT: Computed tomography
RT-PCR: Reverse transcription-polymerase chain reaction
GGO: Ground-glass opacity
NLR: Neutrophil/lymphocyte ratio
ROC: Receiver operating characteristic
AUC: Area under the curve
