# Supplementary material for: A model based on CT radiomic features for predicting RT-PCR becoming negative in coronavirus disease 2019 (COVID-19) patients
Source: BMC Med Imaging. 2020 Oct 20;20:118. doi: 10.1186/s12880-020-00521-z (PMC7573533; doi:10.1186/s12880-020-00521-z)
Supplement: Supplementary file 1 — Additional file 1. Names and definitions of radiomic features. [file 12880_2020_521_MOESM1_ESM.docx]

| **Supplementary data 1: CT quantitative analysis of the RT-PCR-negative and RT-PCR-positive groups.** | | | | | | | | | |
| --- | --- | --- | --- | --- | --- | --- | --- | --- | --- |
| Variable | Total (n=203) | | | Training dataset (n=141) | | | Testing dataset (N=62) | | |
|  | Negative (n=122) | Positive (n=81) | P | Negative (n=85) | Positive (n=56) | p | Negative (n=37) | Positive (n=25) | p |
| **Left upper lobe** | |  |  |  |  |  |  |  |  |
| Lobe volume (mm^3^) | 1064481.00 (873668.60, 1237522.95) | 1069937.00 (876829.40, 1359653.80) | 0.507 | 1028792.00 (872876.60, 1206325.10) | 1085875.00 (880126.40, 1359975.30) | 0.290 | 1131924.16±306741.41 | 1099749.08±273263.77 | 0.674 |
| Lesion volume (mm^3^) | 22.50 (0.00, 3951.25) | 430.00 (0.00, 11872.30) | 0.449 | 7.00 (0.00, 3610.30) | 910.00 (0.00, 14788.85) | 0.137 | 556.00 (0.00, 6794.80) | 128.00 (0.00, 1497.50) | 0.319 |
| Lesion volume  percentage (%) | 0.00 (0.00, 0.40) | 0.00 (0.00, 1.56) | 0.388 | 0.00 (0.00, 0.40) | 0.10 (0.00, 1.75) | 0.091 | 0.00 (0.00, 0.60) | 0.00 (0.00, 0.10) | 0.319 |
| Lesion score | 1.00 (0.00, 1.00) | 1.00 (0.00, 1.00) | 0.813 | 1.00 (0.00, 1.00) | 1.00 (0.00, 1.00) | 0.423 | 1.00 (0.00, 1.00) | 1.00 (0.00, 1.00) | 0.430 |
| Lobe mean CT value (HU) | -803.65 (-823.64,  -780.59) | -810.80 (-826.80,  -785.08) | 0.313 | -802.30 (-822.13,  -775.97) | -810.40 (-826.55,  -772.07) | 0.536 | -805.50 (-825.36,  -786.56) | -812.40 (-827.70,  -795.45) | 0.385 |
| Lobe standard deviation of CT value (HU) | 142.77±10.46 | 142.87±9.61 | 0.941 | 142.39±10.78 | 142.09±10.03 | 0.868 | 143.63±9.78 | 144.63±8.51 | 0.679 |
| Lesion mean CT Value (HU) | -568.75 (-704.37, 0.00) | -611.20 (-716.17, 0.00) | 0.508 | -483.40 (-700.17, 0.00) | -614.80 (-717.75, 0.00) | 0.270 | -618.50 (-709.94, 0.00) | -509.00 (-712.32, 0.00) | 0.610 |
| Lesion standard deviation of CT value (HU) | 46.70 (0.00, 99.99) | 52.80 (0.00, 103.02) | 0.744 | 43.70 (0.00, 92.23) | 58.25 (0.00, 107.89) | 0.379 | 50.80 (0.00, 109.71) | 42.50 (0.00, 93.88) | 0.478 |
| **Left lower lobe** | |  |  |  |  |  |  |  |  |
| Lobe volume (mm^3^) | 925494.00 (733882.95, 1147647.85) | 974051.00 (730893.40, 1191801.90) | 0.598 | 924799.00 (720335.50, 1108030.70) | 929933.00 (679321.45, 1236514.05) | 0.628 | 954334.00 (793095.90, 1209327.40) | 993864.00 (790147.90, 1140566.90) | 0.926 |
| Lesion volume (mm^3^) | 10321.50 (507.80, 48238.80) | 10896.00 (428.40, 52497.20) | 0.770 | 9180.00 (860.60, 47465.60) | 13683.50 (922.40, 63908.05) | 0.431 | 13658.00 (304.50, 48541.40) | 6485.00 (6.30, 44426.50) | 0.505 |
| Lesion volume  percentage (%) | 1.10 (0.00, 5.53) | 1.20 (0.00, 6.85) | 0.743 | 0.80 (0.10, 6.16) | 1.50 (0.10, 9.62) | 0.375 | 1.50 (0.00, 5.40) | 0.70 (0.00, 4.14) | 0.491 |
| Lesion score | 1.00 (1.00, 2.00) | 1.00 (1.00, 2.00) | 0.757 | 1.00 (1.00, 2.00) | 1.00 (1.00, 2.00) | 0.833 | 1.00 (1.00, 2.00) | 1.00 (0.70, 1.30) | 0.366 |
| Lobe mean CT value (HU) | -767.15 (-793.40,  -735.76) | -773.60 (-803.52,  -740.26) | 0.439 | -768.10 (-793.53,  -733.58) | -776.55 (-803.91,  -702.25) | 0.717 | -766.00 (-789.70,  -738.46) | -773.40 (-798.06,  -756.77) | 0.374 |
| Lobe standard deviation of CT value (HU) | 158.12±14.20 | 159.34±11.93 | 0.524 | 157.14±14.88 | 159.19±12.83 | 0.401 | 160.37±12.39 | 159.69±9.84 | 0.819 |
| Lesion mean CT value (HU) | -665.85 (-712.24,  -593.25) | -658.20 (-712.99,  -537.98) | 0.684 | -663.30 (-709.07,  -587.84) | -649.30 (-705.81,  -558.55) | 0.525 | -684.00 (-715.55,  -596.30) | -664.10 (-761.61,  -132.09) | 0.835 |
| Lesion standard deviation of CT value (HU) | 101.05 (53.05, 132.21) | 99.90 (40.58, 133.79) | 0.713 | 105.90 (46.10, 132.98) | 100.80 (50.13, 131.62) | 0.919 | 93.50 (63.91, 131.42) | 75.30 (12.18, 133.79) | 0.385 |
| **Right upper lobe** | |  |  |  |  |  |  |  |  |
| Lobe volume (mm^3^) | 853959.50 (728011.80, 1012095.55) | 893069.00 (735293.10, 1083919.30) | 0.560 | 840461.00 (724899.50, 992484.90) | 908175.00 (740660.90, 1113450.50) | 0.203 | 941484.22±227870.48 | 874294.92±222708.23 | 0.255 |
| Lesion volume (mm^3^) | 340.50 (0.00, 7267.55) | 599.00 (0.00, 8592.10) | 0.747 | 99.00 (0.00, 8295.60) | 885.50 (0.00, 9497.45) | 0.503 | 504.00 (0.00, 6885.90) | 0.00 (0.00, 8223.90) | 0.693 |
| Lesion volume  percentage (%) | 0.00 (0.00, 1.10) | 0.10 (0.00, 1.03) | 0.785 | 0.00 (0.00, 1.20) | 0.15 (0.00, 1.15) | 0.645 | 0.10 (0.00, 0.82) | 0.00 (0.00, 0.80) | 0.758 |
| Lesion score | 1.00 (0.00, 1.00) | 1.00 (0.00, 1.00) | 0.899 | 1.00 (0.00, 1.00) | 1.00 (0.00, 1.00) | 0.622 | 1.00 (0.00, 1.00) | 0.00 (0.00, 1.00) | 0.590 |
| Lobe mean CT value (HU) | -801.00 (-819.61,  -783.99) | -807.00 (-824.48,  -778.84) | 0.452 | -801.30 (-819.46,  -781.91) | -807.65 (-826.91,  -770.49) | 0.437 | -800.70 (-822.07,  -784.14) | -803.00 (-816.79,  -787.79) | 0.920 |
| Lobe standard deviation of CT value (HU) | 139.66±12.15 | 140.13±12.67 | 0.794 | 140.04±12.83 | 138.43±12.39 | 0.462 | 138.79±10.53 | 143.92±12.72 | 0.089 |
| Lesion mean CT value (HU) | -585.55 (-703.36, 0.00) | -650.20 (-720.27, 0.00) | 0.338 | -582.30 (-699.09, 0.00) | -652.50 (-722.63, 0.00) | 0.180 | -608.50 (-707.45, 0.00) | 0.00 (-708.19, 0.00) | 0.791 |
| Lesion standard deviation of CT value (HU) | 47.65 (0.00, 94.05) | 50.60 (0.00, 90.28) | 0.955 | 46.10 (0.00, 99.41) | 54.35 (0.00, 99.12) | 0.728 | 49.20 (0.00, 84.31) | 0.00 (0.00, 71.49) | 0.495 |
| **Right middle lobe** | |  |  |  |  |  |  |  |  |
| Lobe volume (mm^3^) | 383760.00 (330040.90, 464862.25) | 382394.00 (316698.90, 474818.70) | 0.507 | 381220.00 (322579.30, 453700.30) | 384205.50 (322640.80, 469996.80) | 0.853 | 410749.00 (355332.80, 501311.20) | 355095.00 (299888.10, 503178.60) | 0.162 |
| Lesion volume (mm^3^) | 0.00 (0.00, 383.40) | 0.00 (0.00, 1219.50) | 0.324 | 0.00 (0.00, 135.60) | 0.00 (0.00, 1051.20) | 0.363 | 0.00 (0.00, 535.50) | 0.00 (0.00, 1342.80) | 0.641 |
| Lesion volume  percentage (%) | 0.00 (0.00, 0.10) | 0.00 (0.00, 0.30) | 0.351 | 0.00 (0.00, 0.03) | 0.00 (0.00, 0.30) | 0.491 | 0.00 (0.00, 0.13) | 0.00 (0.00, 0.33) | 0.509 |
| Lesion score | 0.00 (0.00, 1.00) | 0.00 (0.00, 1.00) | 0.399 | 0.00 (0.00, 1.00) | 0.00 (0.00, 1.00) | 0.321 | 0.00 (0.00, 1.00) | 0.00 (0.00, 1.00) | 0.977 |
| Lobe mean CT value (HU) | -826.70 (-846.48,  -805.38) | -834.10 (-846.64,  -809.16) | 0.420 | -826.10 (-846.53,  -802.98) | -836.00 (-847.75,  -795.85) | 0.467 | -831.00 (-847.00,  -808.31) | -831.60 (-837.69,  -821.55) | 0.813 |
| Lobe standard deviation of CT value (HU) | 122.25 (114.65, 132.51) | 121.40 (113.99, 134.42) | 0.782 | 123.50±13.91 | 123.75±14.30 | 0.921 | 123.57±14.76 | 124.35±13.03 | 0.833 |
| Lesion mean CT value (HU) | 0.00 (-574.58, 0.00) | 0.00 (-641.53, 0.00) | 0.270 | 0.00 (-560.21, 0.00) | 0.00 (-647.38, 0.00) | 0.299 | 0.00 (-633.09, 0.00) | 0.00 (-641.53, 0.00) | 0.725 |
| Lesion standard deviation of CT value (HU) | 0.00 (0.00, 62.04) | 0.00 (0.00, 85.43) | 0.405 | 0.00 (0.00, 49.43) | 0.00 (0.00, 87.03) | 0.350 | 0.00 (0.00, 87.20) | 0.00 (0.00, 58.59) | 0.994 |
| **Right lower lobe** | |  |  |  |  |  |  |  |  |
| Lobe volume (mm^3^) | 993511.00 (777466.00, 1196354.15) | 975831.00 (790641.60, 1267309.80) | 0.845 | 982970.00 (755718.10, 1176960.50) | 974831.50 (756743.95, 1302151.10) | 0.711 | 1001256.00 (866904.70, 1345686.20) | 1010098.00 (823543.60, 1180919.40) | 0.769 |
| Lesion volume (mm^3^) | 10724.50 (206.10, 67803.70) | 10005.00 (3.50, 96351.40) | 0.839 | 11047.00 (65.10, 69277.50) | 18787.00 (2.25, 121422.25) | 0.637 | 10707.00 (413.70, 65013.10) | 6231.00 (848.40, 70406.30) | 0.813 |
| Lesion volume  percentage (%) | 1.25 (0.00, 6.91) | 1.00 (0.00, 10.10) | 0.696 | 1.30 (0.00, 7.22) | 1.95 (0.00, 14.60) | 0.554 | 1.00 (0.00, 6.46) | 0.50 (0.07, 5.40) | 0.949 |
| Lesion score | 1.00 (1.00, 2.00) | 1.00 (0.70, 2.00) | 0.816 | 1.00 (1.00, 2.00) | 1.00 (0.45, 2.00) | 0.768 | 1.00 (1.00, 2.00) | 1.00 (0.70, 1.30) | 0.336 |
| Lobe mean CT value (HU) | -776.45 (-801.90,  -738.97) | -788.70 (-810.13,  -728.50) | 0.596 | -780.40 (-802.95,  -739.24) | -791.70 (-812.07,  -715.49) | 0.677 | -774.90 (-799.41,  -735.86) | -784.50 (-804.51,  -760.86) | 0.586 |
| Lobe standard deviation of CT value (HU) | 145.70 (136.58, 153.73) | 145.70 (138.65, 156.88) | 0.300 | 144.70 (135.99, 152.20) | 145.30 (137.34, 157.20) | 0.311 | 147.14±13.55 | 147.46±10.49 | 0.920 |
| Lesion mean CT value (HU) | -661.30 (-714.32,  -555.70) | -650.10 (-703.61,  -336.98) | 0.508 | -659.30 (-715.32,  -542.41) | -642.80 (-699.44,  -216.63) | 0.347 | -662.80 (-713.88,  -583.99) | -682.20 (-710.09,  -426.72) | 0.758 |
| Lesion standard deviation of CT value (HU) | 89.15 (40.50, 125.88) | 93.40 (9.87, 120.64) | 0.950 | 89.80 (35.94, 125.38) | 99.40 (6.34, 125.69) | 0.830 | 82.70 (49.08, 129.61) | 93.00 (19.32, 114.70) | 0.880 |
| **Left lung** | |  |  |  |  |  |  |  |  |
| Lobe volume (mm^3^) | 1982490.00 (1601488.50, 2398025.45) | 2039811.00 (1680728.00, 2505494.90) | 0.505 | 1898276.00 (1589260.30, 2308099.00) | 2054599.00 (1588594.25, 2528561.50) | 0.414 | 2034309.00 (1707688.30, 2514566.90) | 1996475.00 (1709632.60, 2459250.80) | 0.892 |
| Lesion volume (mm^3^) | 11394.00 (1331.60, 61190.00) | 17060.00 (975.40, 74689.60) | 0.378 | 11006.00 (1535.60, 50586.20) | 26740.00 (3082.55, 90001.45) | 0.106 | 13658.00 (1222.40, 73255.10) | 6485.00 (279.30, 51338.70) | 0.401 |
| Lesion volume  percentage (%) | 0.60 (0.10, 3.00) | 0.80 (0.07, 3.66) | 0.531 | 0.60 (0.10, 3.00) | 1.15 (0.14, 4.57) | 0.156 | 0.70 (0.07, 3.58) | 0.40 (0.00, 2.02) | 0.344 |
| Lesion score | 2.00 (1.00, 3.00) | 2.00 (1.00, 3.00) | 0.933 | 2.00 (1.00, 3.00) | 2.00 (1.00, 3.00) | 0.526 | 2.00 (1.00, 3.00) | 1.00 (1.00, 2.30) | 0.263 |
| Lobe mean CT value (HU) | -786.55 (-809.63,  -759.17) | -798.80 (-814.91,  -764.50) | 0.390 | -788.00 (-810.23,  -758.51) | -799.35 (-815.73,  -744.60) | 0.657 | -786.10 (-809.53,  -761.23) | -795.20 (-810.08,  -774.84) | 0.397 |
| Lobe standard deviation of CT value (HU) | 151.70±11.98 | 152.58±10.30 | 0.589 | 151.11±12.68 | 152.35±11.18 | 0.553 | 153.06±10.23 | 153.10±8.18 | 0.986 |
| Lesion mean CT Value (HU) | -677.40 (-716.62,  -606.59) | -673.20 (-736.39,  -603.80) | 0.735 | -665.60 (-711.96,  -606.21) | -670.35 (-716.70,  -584.82) | 0.908 | -696.60 (-719.49,  -610.81) | -696.90 (-773.85,  -632.89) | 0.662 |
| Lesion standard deviation of CT value (HU) | 101.75 (61.79, 132.21) | 100.70 (60.62, 133.42) | 0.912 | 103.70 (60.32, 132.98) | 101.15 (70.01, 130.78) | 0.675 | 98.10 (67.98, 124.07) | 84.60 (48.99, 133.82) | 0.551 |
| **Right lung** | |  |  |  |  |  |  |  |  |
| Lobe volume (mm^3^) | 2285886.50 (1836646.65, 2649338.85) | 2250614.00 (1851587.40, 2763112.50) | 0.882 | 2202362.00 (1824425.00, 2585166.80) | 2273184.50 (1849604.55, 2792733.90) | 0.453 | 2298960.00 (2045211.90, 2864380.40) | 2250614.00 (1851299.70, 2624800.90) | 0.370 |
| Lesion volume (mm^3^) | 14776.00 (902.45, 80753.95) | 13121.00 (1636.20, 101622.70) | 0.717 | 14487.00 (949.20, 79827.90) | 22586.00 (767.20, 169029.60) | 0.564 | 16796.00 (660.60, 94320.10) | 8527.00 (1967.80, 80269.30) | 0.846 |
| Lesion volume  percentage (%) | 0.60 (0.00, 4.01) | 0.60 (0.10, 5.03) | 0.661 | 0.70 (0.00, 3.91) | 0.85 (0.00, 7.07) | 0.558 | 0.60 (0.00, 4.06) | 0.30 (0.10, 3.24) | 1.000 |
| Lesion score | 2.00 (1.00, 3.00) | 2.00 (1.00, 3.30) | 0.923 | 2.00 (1.00, 3.00) | 2.00 (1.00, 4.00) | 0.604 | 2.00 (1.00, 4.00) | 2.00 (1.00, 3.00) | 0.495 |
| Lobe mean CT value (HU) | -796.85 (-815.74,  -771.06) | -804.50 (-820.10,  -766.11) | 0.469 | -796.70 (-815.94,  -772.22) | -806.90 (-823.74,  -753.80) | 0.515 | -797.00 (-813.67,  -770.17) | -802.20 (-814.53,  -782.66) | 0.636 |
| Lobe standard deviation of CT value (HU) | 141.00 (132.70, 150.92) | 143.90 (134.05, 153.33) | 0.314 | 141.00 (132.40, 150.99) | 143.35 (133.12, 154.08) | 0.545 | 141.98±10.97 | 144.12±9.54 | 0.431 |
| Lesion mean CT value (HU) | -678.85 (-720.04,  -606.29) | -690.60 (-737.88,  -614.45) | 0.564 | -674.30 (-727.83,  -623.19) | -674.80 (-735.43,  -584.78) | 0.858 | -680.60 (-715.68,  -601.55) | -700.40 (-740.76,  -627.95) | 0.401 |
| Lesion standard deviation of CT value (HU) | 95.60 (59.98, 125.82) | 95.50 (57.39, 126.68) | 0.930 | 95.20 (58.50, 126.42) | 99.10 (58.16, 127.26) | 0.781 | 93.93±51.97 | 85.42±52.52 | 0.531 |
| **Whole lung** | |  |  |  |  |  |  |  |  |
| Lobe volume (mm^3^) | 4214841.00 (3457069.50, 5046459.65) | 4265631.00 (3504580.60, 5195166.90) | 0.693 | 4182753.00 (3415720.60, 4853447.90) | 4224215.00 (3516524.80, 5327626.90) | 0.431 | 4392879.00 (3850303.10, 5366880.10) | 4322388.00 (3468583.20, 5052184.30) | 0.581 |
| Lesion volume (mm^3^) | 35910.50 (5061.75, 126801.15) | 48967.00 (8189.80, 173299.90) | 0.386 | 34410.00 (5030.50, 118071.90) | 66305.00 (10370.85, 247135.35) | 0.166 | 40959.00 (5345.30, 169111.60) | 19558.00 (3855.00, 118827.40) | 0.610 |
| Lesion volume  percentage (%) | 0.85 (0.10, 3.21) | 1.00 (0.17, 4.73) | 0.523 | 0.80 (0.10, 3.29) | 1.45 (0.30, 5.71) | 0.248 | 1.10 (0.14, 3.68) | 0.60 (0.10, 2.15) | 0.556 |
| Lesion score | 4.00 (2.00, 6.00) | 4.00 (2.00, 6.00) | 0.962 | 3.00 (2.00, 5.00) | 4.00 (2.00, 6.55) | 0.487 | 4.00 (2.00, 6.30) | 3.00 (1.70, 5.00) | 0.305 |
| Lobe mean CT value (HU) | -791.55 (-813.62,  -767.10) | -802.60 (-817.52,  -766.26) | 0.430 | -792.80 (-813.69,  -767.87) | -802.80 (-819.32,  -750.49) | 0.580 | -788.90 (-808.98,  -766.50) | -795.70 (-813.26,  -779.82) | 0.482 |
| Lobe standard deviation of CT value (HU) | 146.05 (138.60, 154.72) | 148.00 (140.97, 157.59) | 0.280 | 145.50 (136.90, 153.90) | 147.05 (140.03, 158.36) | 0.367 | 147.35±10.08 | 148.46±8.26 | 0.649 |
| Lesion mean CT value (HU) | -699.55 (-728.80,  -647.00) | -700.70 (-770.20,  -648.52) | 0.245 | -688.77±68.57 | -697.74±92.32 | 0.535 | -700.84±69.81 | -722.64±63.25 | 0.215 |
| Lesion standard deviation of CT value (HU) | 102.88±38.00 | 105.40±43.70 | 0.664 | 104.55±38.86 | 107.06±45.55 | 0.727 | 99.05±36.18 | 101.70±39.88 | 0.788 |
| **Airway** | |  |  |  |  |  |  |  |  |
| Lung volume (mm^3^) | 74887.50 (65893.95, 91010.65) | 77444.00 (65577.80, 94526.00) | 0.498 | 73693.00 (65328.20, 91053.90) | 77713.00 (66380.25, 96334.95) | 0.328 | 79517.00 (71172.60, 90243.40) | 72416.00 (61916.20, 93173.70) | 0.824 |
| Mean CT value (HU) | -541.26±47.48 | -546.01±49.40 | 0.494 | -541.57±46.37 | -544.17±53.44 | 0.760 | -540.55±50.59 | -550.13±39.55 | 0.429 |
| Standard deviation  of CT value (HU) | 336.10 (326.46, 371.59) | 359.00 (328.10, 375.80) | 0.106 | 336.50 (326.71, 372.04) | 344.30 (326.74, 375.58) | 0.478 | 335.40 (323.94, 371.99) | 368.70 (330.50, 379.12) | 0.095 |
| **Total Score** | 4.00 (2.00, 5.05) | 4.00 (2.00, 6.00) | 0.889 | 3.00 (2.00, 5.00) | 4.00 (2.00, 6.55) | 0.422 | 4.00 (2.00, 6.30) | 3.00 (1.70, 5.00) | 0.305 |
| **Part solid** | |  |  |  |  |  |  |  |  |
| Percentage in left lung (%) | 0.60 (0.10, 3.00) | 0.80 (0.07, 3.63) | 0.540 | 0.60 (0.10, 2.93) | 1.15 (0.14, 4.41) | 0.165 | 0.70 (0.07, 3.48) | 0.40 (0.00, 2.02) | 0.351 |
| Percentage in right lung (%) | 0.60 (0.00, 3.91) | 0.60 (0.10, 5.03) | 0.659 | 0.70 (0.00, 3.88) | 0.85 (0.00, 6.96) | 0.558 | 0.60 (0.00, 3.99) | 0.30 (0.10, 3.24) | 0.989 |
| Percentage in whole lung (%) | 0.85 (0.10, 3.21) | 1.00 (0.17, 4.70) | 0.512 | 0.80 (0.10, 3.29) | 1.45 (0.30, 5.61) | 0.238 | 1.10 (0.14, 3.55) | 0.60 (0.10, 2.08) | 0.561 |
| Volume in left lung (mm^3^) | 11367.50 (1331.00, 58786.15) | 17049.00 (975.10, 74450.10) | 0.380 | 10804.00 (1532.00, 50136.50) | 24986.50 (3081.45, 89993.35) | 0.105 | 13610.00 (1222.40, 72944.10) | 6485.00 (279.30, 50341.60) | 0.417 |
| Volume in right lung (mm^3^) | 14748.50 (911.95, 80601.35) | 13201.00 (1633.50, 101379.90) | 0.688 | 14456.00 (952.20, 79552.00) | 22461.00 (749.65, 166374.30) | 0.538 | 16744.00 (660.60, 92554.60) | 9146.00 (1965.00, 79969.40) | 0.880 |
| Volume in whole lung (mm^3^) | 35795.50 (5053.20, 125943.40) | 51024.00 (8120.10, 180938.50) | 0.323 | 34446.00 (5024.20, 117566.70) | 69799.50 (10380.80, 261737.55) | 0.128 | 40846.00 (5345.30, 166309.60) | 19881.00 (3871.10, 117490.50) | 0.610 |
| Mean CT value in left lung (HU) | -685.75 (-719.55,  -616.05) | -674.70 (-737.12,  -610.49) | 0.713 | -667.40 (-713.66,  -611.86) | -672.00 (-717.76,  -596.48) | 0.886 | -701.40 (-720.92,  -634.81) | -698.90 (-773.85,  -648.36) | 0.621 |
| Mean CT value in right lung (HU) | -683.45 (-721.49,  -614.49) | -694.70 (-741.45,  -623.81) | 0.508 | -675.30 (-728.39,  -632.04) | -682.95 (-735.78,  -598.57) | 0.868 | -686.40 (-715.98,  -607.07) | -706.70 (-746.08,  -643.72) | 0.366 |
| Mean CT value in whole lung (HU) | -701.75 (-730.75,  -651.38) | -702.10 (-770.20,  -656.99) | 0.250 | -698.40 (-733.75,  -649.31) | -697.00 (-783.79,  -643.84) | 0.618 | -704.54±66.38 | -726.99±58.75 | 0.177 |
| **Solid** | |  |  |  |  |  |  |  |  |
| Percentage in left lung (%) | 0.00 (0.00, 0.00) | 0.00 (0.00, 0.00) | 0.286 | 0.00 (0.00, 0.00) | 0.00 (0.00, 0.10) | 0.154 | 0.00 (0.00, 0.00) | 0.00 (0.00, 0.00) | 0.818 |
| Percentage in right lung (%) | 0.00 (0.00, 0.00) | 0.00 (0.00, 0.00) | 0.615 | 0.00 (0.00, 0.00) | 0.00 (0.00, 0.00) | 0.569 | 0.00 (0.00, 0.00) | 0.00 (0.00, 0.00) | 0.966 |
| Percentage in whole lung (%) | 0.00 (0.00, 0.00) | 0.00 (0.00, 0.00) | 0.466 | 0.00 (0.00, 0.00) | 0.00 (0.00, 0.10) | 0.243 | 0.00 (0.00, 0.00) | 0.00 (0.00, 0.00) | 0.636 |
| Volume in left lung (mm^3^) | 55.00 (0.00, 414.35) | 40.00 (0.00, 625.20) | 0.916 | 55.00 (0.00, 416.10) | 107.50 (0.00, 871.95) | 0.404 | 55.00 (0.00, 426.20) | 5.00 (0.00, 245.00) | 0.319 |
| Volume in right lung (mm^3^) | 24.50 (0.00, 315.40) | 10.00 (0.00, 279.20) | 0.980 | 28.00 (0.00, 328.40) | 18.50 (0.00, 389.50) | 0.953 | 20.00 (0.00, 132.50) | 9.00 (0.00, 242.80) | 0.960 |
| Volume in whole lung (mm^3^) | 140.50 (0.95, 826.00) | 111.00 (1.70, 1637.70) | 0.975 | 131.00 (1.00, 841.00) | 151.00 (1.45, 2000.90) | 0.752 | 160.00 (0.00, 876.40) | 58.00 (2.10, 487.80) | 0.662 |
| Mean CT value in left lung (HU) | -128.95 (-149.64, 0.00) | -112.40 (-134.16, 0.00) | 0.040 | -131.90 (-150.49, 0.00) | -117.15 (-136.66, 0.00) | 0.281 | -124.50 (-146.80, 0.00) | -83.20 (-127.68, 0.00) | 0.042 |
| Mean CT value in right lung (HU) | -115.85 (-142.81, 0.00) | -102.40 (-140.04, 0.00) | 0.367 | -117.10 (-142.45, 0.00) | -102.35 (-142.42, 0.00) | 0.574 | -113.20 (-143.47, 0.00) | -103.10 (-129.40, 0.00) | 0.401 |
| Mean CT value in whole lung (HU) | -129.15 (-148.17,  -71.82) | -117.60 (-140.48,  -66.45) | 0.294 | -131.60 (-150.94,  -83.30) | -117.80 (-139.31,  -81.74) | 0.272 | -122.60 (-137.87, 0.00) | -110.20 (-142.70,  -24.71) | 0.791 |

Quantitative variables that obey normal distribution are presented as the mean± standard deviation, while those who do not obey normal distribution are shown as median (25% percentile, 75% percentile).

**Supplementary data 2: Definition of radiomic features**

**(Adapted from** [**http://pyradiomics.readthedocs.io/en/latest/features.html**](http://pyradiomics.readthedocs.io/en/latest/features.html) **– last accessed June 1, 2020)**

***a. First Order Features***

These statistics describe the central tendency, variability, uniformity, asymmetry, skewness and magnitude of the attenuation values in a given region of interest (ROI), disregarding the spatial relationship of the individual voxels. As such, they describe quantitative and qualitative features of the whole ROI. A total of 19 features were calculated for the original CT image (Table S1), as follows:

**Let:**

- ***X*** be a set of ***N_p_*** voxels included in the region of interest (ROI)
- ***P(i)*** be the first order histogram with ***N_g_***discrete intensity levels, where ***N_g_*** is the number of non-zero bins, equally spaced from 0 with a width.
- ***p(i)*** be the normalized first order histogram and equal to $\frac{\text{P}\boldsymbol{(i)}}{\boldsymbol{N}_{\boldsymbol{p}}}$
- **c** is a value that shifts the intensities to prevent negative values in X. This ensures that voxels with the lowest gray values contribute the least to Energy, instead of voxels with gray level intensity closest to 0.
- ***ϵ*** is an arbitrarily small positive number (≈2.2×10^−16^)

| **Table S1. First-order features** | |
| --- | --- |
| **Radiomic feature** | **Interpretation** |
| $\mathbf{E}\text{nergy}=\sum_{i=1}^{N_{p}} (\text{X}(i)+c)^{2}$ | **Energy** is a measure of the magnitude of voxel values in an image. A larger value implies a greater sum of the squares of these values. |
| $\text{Total Energy}=V_{voxel}\sum_{i=1}^{N_{p}} (\text{X}(i)+c)^{2}$ | **Total Energy** is the value of Energy feature scaled by the volume of the voxel in cubic mm. |
| $\text{Entropy}=-\sum_{i=1}^{N_{g}} p(i)\log_{2}(p(i)+\epsilon)$ | **Entropy** specifies the uncertainty/randomness in the image values. It measures the average amount of information required to encode the image values |
| $\text{Minimum}=min(\text{X})$ | The **minimum** gray level intensity within the ROI. |
| The **10th percentile** of X | The **10th percentile** of X |
| The **90th percentile** of X | The **90th percentile** of X |
| $\text{Maximum}=max(\text{X})$ | The **maximum** gray level intensity within the ROI. |
| $\text{Mean}=\frac{1}{N_{p}}\sum_{i=1}^{N_{p}} \text{X}(i)$ | The average (**mean**) gray level intensity within the ROI. |
| **Median** | The **median** gray level intensity within the ROI. |
| $\text{Interquartile range}=\text{P}_{75}-\text{P}_{25}$ | Here P_25_ and P_75_ are the 25^th^ and 75^th^ percentile of the image array, respectively. |
| $\text{Range}=max(\text{X})-min(\text{X})$ | The range of gray values in the ROI. |
| $\text{MAD}=\frac{1}{N_{p}}\sum_{i=1}^{N_{p}} \vert\text{X}(i)-\overline{X}\vert$ | **Mean Absolute Deviation (MAD)** is the mean distance of all intensity values from the Mean Value of the image array. |
| $\text{rMAD}=\frac{1}{N_{10-90}}\sum_{i=1}^{N_{10-90}} \vert\text{X}_{10-90}(i)-\overline{X}_{10-90}\vert$ | **Robust Mean Absolute Deviation (rMAD)** is the mean distance of all intensity values from the Mean Value calculated on the subset of image array with gray levels in between, or equal to the 10^th^ and 90^th^ percentile. |
| $\text{RMS}=\sqrt{\frac{1}{N_{p}}\sum_{i=1}^{N_{p}} (\text{X}(i)+c)^{2}}$ | **Root Mean Squared (RMS)** is the square-root of the mean of all the squared intensity values. It is another measure of the magnitude of the image values. This feature is volume-confounded, a larger value of c increases the effect of volume-confounding. |
| $\boldsymbol{SD}=\sqrt{\frac{1}{N_{p}}\sum_{i=1}^{N_{p}} (\text{X}\left( i \right)-{\overline{X})}^{2}}$ | **Standard Deviation (SD)** measures the amount of variation or dispersion from the Mean Value. By definition, standard deviation=$\sqrt{variance}$ |
| $\text{Skewness}=\frac{\mu_{3}}{\sigma^{3}}=\frac{\frac{1}{N_{p}}\sum_{i=1}^{N_{p}} (\text{X}(i)-\overline{X})^{3}}{{(\sqrt{\frac{1}{N_{p}}\sum_{i=1}^{N_{p}} (\text{X}(i)-\overline{X})^{2}})}^{3}}$ | **Skewness** measures the *asymmetry* of the distribution of values about the Mean value. Depending on where the tail is elongated and the mass of the distribution is concentrated, this value can be positive or negative. (Where μ^3^ is the 3^rd^ central moment). |
| $\text{Kurtosis}=\frac{\mu_{4}}{\sigma^{4}}=\frac{\frac{1}{N_{p}}\sum_{i=1}^{N_{p}} (\text{X}(i)-\overline{X})^{4}}{{(\frac{1}{N_{p}}\sum_{i=1}^{N_{p}} (\text{X}(i)-\overline{X})^{2})}^{2}}$ | **Kurtosis** is a measure of the ‘*peakedness*’ of the distribution of values in the image ROI. A higher kurtosis implies that the mass of the distribution is concentrated towards the tail(s) rather than towards the mean. A lower kurtosis implies the reverse: that the mass of the distribution is concentrated towards a spike near the Mean value. (Where μ4 is the 4th central moment). |
| $\text{Variance}=\frac{1}{N_{p}}\sum_{i=1}^{N_{p}} (\text{X}(i)-\overline{X})^{2}$ | **Variance** is the the mean of the squared distances of each intensity value from the Mean value. This is a measure of the spread of the distribution about the mean. |
| $\text{Uniformity}=\sum_{i=1}^{N_{g}} p(i)^{2}$ | **Uniformity** is a measure of the sum of the squares of each intensity value. This is a measure of the heterogeneity of the image array, ***where a greater uniformity implies a greater heterogeneity or a greater range of discrete intensity values****.* |

***b. 2D and 3D Shape Features***

Shape features describe the size and shape of a given ROI, without taking into account the attenuation values of its voxels. Since they are independent of the gray level intensities, shape features were consistent across all wavelet transformation and the original CT image, and therefore were only calculated once. These were defined as follows (Table S2):

Let:

**V** the volume of the ROI in mm^3^

**A** the surface area of the ROI in mm^2^

| **Table S2. 2D and 3D shape features** | |
| --- | --- |
| **Radiomic feature** | **Interpretation** |
| $\mathbf{Volume}=\sum_{i=1}^{N} V_{i}$ | The **volume** of the ROI V is approximated by multiplying the number of voxels in the ROI by the volume of a single voxel **V_i_**. |
| $\mathbf{Surface Area}=\sum_{i=1}^{N} \frac{1}{2}\vert\text{a}_{i}\text{b}_{i}\times\text{a}_{i}\text{c}_{i}\vert$ | **Surface Area** is an approximation of the surface of the ROI in mm^2^, calculated using a marching cubes algorithm, where ***N*** is the number of triangles forming the surface mesh of the volume (ROI), ***a_i_b_i_*** and ***a_i_c_i_*** are the edges of the ***i^th^*** triangle formed by points ***a_i_, b_i_*** and ***c_i._*** |
| $\text{Surface to volume ratio}=\frac{A}{V}$ | Here, a lower value indicates a more compact (sphere-like) shape. This feature is not dimensionless, and is therefore (partly) dependent on the volume of the ROI. |
| $\text{Sphericity}=\frac{\sqrt[3]{36\pi V^{2}}}{A}$ | **Sphericity** is a measure of the roundness of the shape of the tumor region relative to a sphere. It is a dimensionless measure, independent of scale and orientation. The value range is 0<sphericity≤1, where a value of 1 indicates a perfect sphere (a sphere has the smallest possible surface area for a given volume, compared to other solids). |
| **Volume Number** | Total number of discrete volumes in the ROI. |
| **Voxel Number** | Total number of discrete voxels in the ROI. |
| **Maximum 3D diameter** | Maximum 3D diameter is defined as the largest pairwise Euclidean distance between surface voxels in the ROI (Feret Diameter). |
| **Maximum 2D diameter (Slice)** | Maximum 2D diameter (Slice) is defined as the largest pairwise Euclidean distance between ROI surface voxels in the row-column (generally the axial) plane. |
| **Maximum 2D diameter (Column)** | Maximum 2D diameter (Column) is defined as the largest pairwise Euclidean distance between ROI surface voxels in the row-slice (usually the coronal) plane. |
| **Maximum 2D diameter (Row)** | Maximum 2D diameter (Row) is defined as the largest pairwise Euclidean distance between tumor surface voxels in the column-slice (usually the sagittal) plane. |
| $\text{Major axis}=4\sqrt{\lambda_{\text{major}}}$ |  |
| $\text{Minor axis}=4\sqrt{\lambda_{\text{minor}}}$ |  |
| $\text{Least axis}=4\sqrt{\lambda_{\text{least}}}$ |  |
| $\text{Elongation}=\sqrt{\frac{\lambda_{\text{minor}}}{\lambda_{\text{major}}}}$ | Here, **λ_major_** and **λ_minor_** are the lengths of the largest and second largest principal component axes. The values range between 1 (circle-like (non-elongated)) and 0 (single point or 1 dimensional line). |
| $\text{Flatness}=\sqrt{\frac{\lambda_{\text{least}}}{\lambda_{\text{major}}}}$ | Here, **λ_major_** and **λ_minor_** are the lengths of the largest and smallest principal component axes. The values range between 1 (non-flat, sphere-like) and 0 (a flat object). |
|  | |

***c. Gray Level Co-occurrence Matrix (GLCM)***

In simple words, a GLCM describes the number of times a voxel of a given attenuation value ***i*** is located next to a voxel of ***j***. A GLCM of size ***N_g_***×***N_g_*** describes the second-order joint probability function of an image region constrained by the mask and is defined as P(***i,j***|***δ,θ***). The (***i,j***)^th^ element of this matrix represents the number of times the combination of levels ***i*** and ***j*** occur in two pixels in the image, that are separated by a distance of ***δ*** pixels along angle ***θ***. The distance ***δ*** from the center voxel is defined as the distance according to the infinity norm. For ***δ=1***, this results in 2 neighbors for each of 13 angles in 3D (26-connectivity) and for ***δ=2*** a 98-connectivity (49 unique angles). In order to get rotationally invariant results, statistics are calculated in all directions and then averaged, to ensure a symmetrical GLCM (Table S3).

Let:

**ϵ** be an arbitrarily small positive number **(≈2.2×10−16**)

**P(*i,j*)** be the co-occurence matrix for an arbitrary ***δ*** and ***θ***

**p(*i,j*)** be the normalized co-occurence matrix and equal to $\frac{\text{P}(i,j)}{\sum\text{P}(i,j)}$

Ng be the number of discrete intensity levels in the image

$\boldsymbol{p}_{\boldsymbol{x}}\boldsymbol{(i)=}\sum_{\boldsymbol{j=1}}^{\boldsymbol{N}_{\boldsymbol{g}}} \boldsymbol{P(i,j)}$ be the marginal row probabilities

$\boldsymbol{p}_{\boldsymbol{y}}\boldsymbol{(j)=}\sum_{\boldsymbol{i=1}}^{\boldsymbol{N}_{\boldsymbol{g}}} \boldsymbol{P(i,j)}$ be the marginal column probabilities

***μ_x_*** be the mean gray level intensity of ***p_x_*** and defined as $\boldsymbol{\mu}_{\boldsymbol{x}}\boldsymbol{=}\sum_{\boldsymbol{i=1}}^{\boldsymbol{N}_{\boldsymbol{g}}} \boldsymbol{p}_{\boldsymbol{x}}\boldsymbol{(i)i}$

***μ_y_*** be the mean gray level intensity of ***p_y_*** and defined as $\boldsymbol{\mu}_{\boldsymbol{y}}\boldsymbol{=}\sum_{\boldsymbol{j=1}}^{\boldsymbol{N}_{\boldsymbol{g}}} \boldsymbol{p}_{\boldsymbol{y}}\boldsymbol{(j)j}$

***σ_x_*** be the standard deviation of ***p_x_***

***σ_y_*** be the standard deviation of ***p_y_***

$$p_{x+y}(k)=\sum_{i=1}^{N_{g}} \sum_{j=1}^{N_{g}} p(i,j),\text{ where }i+j=k,\text{ and }k=2,3,\ldots,2N_{g}$$

$$p_{x-y}(k)=\sum_{i=1}^{N_{g}} \sum_{j=1}^{N_{g}} p(i,j),\text{ where }|i-j|=k,\text{ and }k=0,1,\ldots,N_{g}-1$$

$HX=-\sum_{i=1}^{N_{g}} p_{x}(i)\log_{2}(p_{x}(i)+\epsilon)$ be the entropy of ***p_x_***

$HY=-\sum_{j=1}^{N_{g}} p_{y}(j)\log_{2}(p_{y}(j)+\epsilon)$ be the entropy of ***p_y_***

$$HXY1=-\sum_{i=1}^{N_{g}} \sum_{j=1}^{N_{g}} p(i,j)\log_{2}(p_{x}(i)p_{y}(j)+\epsilon)$$

$$HXY2=-\sum_{i=1}^{N_{g}} \sum_{j=1}^{N_{g}} p_{x}(i)p_{y}(j)\log_{2}(p_{x}(i)p_{y}(j)+\epsilon)$$

For distance weighting, GLCM matrices are weighted by weighting factor W and then summed and normalised.. Weighting factor W is calculated for the distance between neighbouring voxels by $\boldsymbol{W=}\boldsymbol{e}^{\boldsymbol{-\|d}\boldsymbol{\|}^{\boldsymbol{2}}}$, where d is the distance for the associated angle.

| **Table S3. Gray Level Co-occurrence Matrix (GLCM)** | |
| --- | --- |
| **Radiomic feature** | **Interpretation** |
| $\text{Autocorrelation}=\sum_{i=1}^{N_{g}} \sum_{j=1}^{N_{g}} p(i,j)ij$ | **Autocorrelation** is a measure of the magnitude of the fineness and coarseness of texture. |
| $\text{Joint average}=\mu_{x}=\sum_{i=1}^{N_{g}} \sum_{j=1}^{N_{g}} p(i,j)i$ | Returns the mean gray level intensity of the i distribution. |
| $\text{Cluster prominence}=\sum_{i=1}^{N_{g}} \sum_{j=1}^{N_{g}} (i+j-\mu_{x}-\mu_{y})^{4}p(i,j)$ | **Cluster Prominence** is a measure of the skewness and asymmetry of the GLCM. A higher value implies more asymmetry around the mean while a lower value indicates a peak near the mean value and less variation around the mean. |
| $\text{Cluster tendency}=\sum_{i=1}^{N_{g}} \sum_{j=1}^{N_{g}} (i+j-\mu_{x}-\mu_{y})^{2}p(i,j)$ | **Cluster Tendency** is a measure of groupings of voxels with similar gray-level values. |
| $\text{Cluster shade}=\sum_{i=1}^{N_{g}} \sum_{j=1}^{N_{g}} (i+j-\mu_{x}-\mu_{y})^{3}p(i,j)$ | **Cluster Shade** is a measure of the skewness and uniformity of the GLCM. A higher cluster shade implies greater asymmetry about the mean. |
| $\text{Contrast}=\sum_{i=1}^{N_{g}} \sum_{j=1}^{N_{g}} (i-j)^{2}p(i,j)$ | **Contrast** is a measure of the local intensity variation, favoring values away from the diagonal (i=j). A larger value correlates with a greater disparity in intensity values among neighboring voxels. |
| $\text{Correlation}=\frac{\sum_{i=1}^{N_{g}} \sum_{j=1}^{N_{g}} p(i,j)ij-\mu_{x}\mu_{y}}{\sigma_{x}(i)\sigma_{y}(j)}$ | **Correlation** is a value between 0 (uncorrelated) and 1 (perfectly correlated) showing the linear dependency of gray level values to their respective voxels in the GLCM. |
| $\text{Difference average}=\sum_{k=0}^{N_{g}-1} kp_{x-y}(k)$ | **Difference Average** measures the relationship between occurrences of pairs with similar intensity values and occurrences of pairs with differing intensity values. |
| $\text{Difference entropy}=\sum_{k=0}^{N_{g}-1} p_{x-y}(k)\log_{2}(p_{x-y}(k)+\epsilon)$ | **Difference Entropy** is a measure of the randomness/variability in neighborhood intensity value differences. |
| $\text{Difference variance}=\sum_{k=0}^{N_{g}-1} (k-DA)^{2}p_{x-y}(k)$ | **Difference Variance** is a measure of heterogeneity that places higher weights on differing intensity level pairs that deviate more from the mean. |
| $\text{Joint energy}=\sum_{i=1}^{N_{g}} \sum_{j=1}^{N_{g}} (p(i,j))^{2}$ | **Joint energy** is a measure of homogeneous patterns in the image. A greater **joint energy** implies that there are more instances of intensity value pairs in the image that neighbor each other at higher frequencies. |
| $\text{Joint entropy}=-\sum_{i=1}^{N_{g}} \sum_{j=1}^{N_{g}} p(i,j)\log_{2}(p(i,j)+\epsilon)$ | **Joint entropy** is a measure of the randomness/variability in neighborhood intensity values. |
| $\text{IMC 1}=\frac{HXY-HXY1}{\max\left\{ HX,HY \right\}}$ | **Informational measure of correlation 1** |
| $\text{IMC 2}=\sqrt{1-e^{-2(HXY2-HXY)}}$ | **Informational measure of correlation 2** |
| $\text{IDM}=\sum_{i=1}^{N_{g}} \sum_{j=1}^{N_{g}} \frac{p(i,j)}{1+\vert i-j\vert^{2}}$ | IDM (**inverse difference moment** a.k.a Homogeneity 2) is a measure of the local homogeneity of an image. IDM weights are the inverse of the Contrast weights (decreasing exponentially from the diagonal i=j in the GLCM). |
| $\text{IDMN}=\sum_{i=1}^{N_{g}} \sum_{j=1}^{N_{g}} \frac{p(i,j)}{1+(\frac{\vert i-j\vert^{2}}{N_{g}^{2}})}$ | IDMN (**inverse difference moment normalized**) is a measure of the local homogeneity of an image. IDMN weights are the inverse of the Contrast weights (decreasing exponentially from the diagonal i=j in the GLCM). Unlike Homogeneity2, IDMN normalizes the square of the difference between neighboring intensity values by dividing over the square of the total number of discrete intensity values. |
| $\text{ID}=\sum_{i=1}^{N_{g}} \sum_{j=1}^{N_{g}} \frac{p(i,j)}{1+\vert i-j\vert}$ | ID (**inverse difference** a.k.a. Homogeneity 1) is another measure of the local homogeneity of an image. With more uniform gray levels, the denominator will remain low, resulting in a higher overall value. |
| $\text{IDN}=\sum_{i=1}^{N_{g}} \sum_{j=1}^{N_{g}} \frac{p(i,j)}{1+(\frac{\vert i-j\vert}{N_{g}})}$ | IDN (**inverse difference normalized**) is another measure of the local homogeneity of an image. Unlike Homogeneity1, IDN normalizes the difference between the neighboring intensity values by dividing over the total number of discrete intensity values. |
| $\text{Inverse variance}=\sum_{i=1}^{N_{g}} \sum_{j=1}^{N_{g}} \frac{p(i,j)}{\vert i-j\vert^{2}},i\neq j$ |  |
| $\text{Maximum probability}=max(p(i,j))$ | **Maximum Probability** is occurrences of the most predominant pair of neighboring intensity values. |
| $\text{Sum average}=\sum_{k=2}^{2N_{g}} p_{x+y}(k)k$ | **Sum Average** measures the relationship between occurrences of pairs with lower intensity values and occurrences of pairs with higher intensity values. |
| $\text{Sum entropy}=\sum_{k=2}^{2N_{g}} p_{x+y}(k)\log_{2}(p_{x+y}(k)+\epsilon)$ | **Sum Entropy** is a sum of neighborhood intensity value differences. |
| $\text{Sum squares}=\sum_{i=1}^{N_{g}} \sum_{j=1}^{N_{g}} (i-\mu_{x})^{2}p(i,j)$ | **Sum of Squares** or Variance is a measure in the distribution of neigboring intensity level pairs about the mean intensity level in the GLCM. |
|  | |

***d. Gray Level Run Length Matrix (GLRLM)***

A Gray Level Run Length Matrix (GLRLM) describes gray level runs, which are defined as the length in number of pixels, of consecutive pixels that have the same gray level value. In a gray level run length matrix ***P(i,j|θ)***, the ***(i,j)^th^*** element describes the number of runs with gray level ***i*** and length ***j*** occur in the image (ROI) along angle ***θ*** (Table S4).

Let:

***N_g_*** be the number of discreet intensity values in the image

***N_r_*** be the number of discreet run lengths in the image

***N_p_*** be the number of voxels in the image

***N_z_(θ)*** be the number of runs in the image along angle θ, which is equal to $\sum_{i=1}^{N_{g}} \sum_{j=1}^{N_{r}} \text{P}(i,j|\theta)$and 1≤N_z_(θ)≤N_p_

***P(i,j|θ)*** be the run length matrix for an arbitrary direction θ

***p(i,j|θ)*** be the normalized run length matrix, defined as $p(i,j|\theta)=\frac{\text{P}(i,j|\theta)}{N_{z}(\theta)}$

**ϵ** is an arbitrarily small positive number (≈2.2×10−16).

By default, the value of a feature is calculated on the GLRLM for each angle separately, after which the mean of these values is returned. If distance weighting is enabled, GLRLMs are weighted by the distance between neighbouring voxels and then summed and normalised. Features are then calculated on the resultant matrix. The distance between neighbouring voxels is calculated for each angle using the norm specified in ‘weightingNorm’

| **Table S4. Gray Level Run Length Matrix (GLRLM)** | |
| --- | --- |
| **Radiomic feature** | **Interpretation** |
| $\text{SRE}=\frac{\sum_{i=1}^{N_{g}} \sum_{j=1}^{N_{r}} \frac{\text{P}(i,j\vert\theta)}{j^{2}}}{N_{z}(\theta)}$ | **SRE (Short Run Emphasis)** is a measure of the distribution of short run lengths, with a greater value indicative of shorter run lengths and more fine textural textures. |
| $\text{LRE}=\frac{\sum_{i=1}^{N_{g}} \sum_{j=1}^{N_{r}} \text{P}(i,j\vert\theta)j^{2}}{N_{z}(\theta)}$ | **LRE (Long Run Emphasis)** is a measure of the distribution of long run lengths, with a greater value indicative of longer run lengths and more coarse structural textures. |
| $\text{GLN}=\frac{\sum_{i=1}^{N_{g}} {(\sum_{j=1}^{N_{r}} \text{P}(i,j\vert\theta))}^{2}}{N_{z}(\theta)}$ | **GLN (Gray Level Non-uniformity)** measures the similarity of gray-level intensity values in the image, where a lower GLN value correlates with a greater similarity in intensity values. |
| $\text{GLNN}=\frac{\sum_{i=1}^{N_{g}} {(\sum_{j=1}^{N_{r}} \text{P}(i,j\vert\theta))}^{2}}{N_{z}(\theta)^{2}}$ | **GLNN (Gray Level Non-uniformity Normalized)** measures the similarity of gray-level intensity values in the image, where a lower GLNN value correlates with a greater similarity in intensity values. This is the normalized version of the GLN formula. |
| $\text{RLN}=\frac{\sum_{j=1}^{N_{r}} {(\sum_{i=1}^{N_{g}} \text{P}(i,j\vert\theta))}^{2}}{N_{z}(\theta)}$ | **RLN (Run Length Non-uniformity)** measures the similarity of run lengths throughout the image, with a lower value indicating more homogeneity among run lengths in the image. |
| $\text{RLNN}=\frac{\sum_{j=1}^{N_{r}} {(\sum_{i=1}^{N_{g}} \text{P}(i,j\vert\theta))}^{2}}{N_{z}(\theta)^{2}}$ | **RLNN (Run Length Non-uniformity)** measures the similarity of run lengths throughout the image, with a lower value indicating more homogeneity among run lengths in the image. This is the normalized version of the RLN formula. |
| $\text{RP}=\frac{N_{z}(\theta)}{N_{p}}$ | **RP (Run Percentage)** measures the coarseness of the texture by taking the ratio of number of runs and number of voxels in the ROI. Values are in range $\frac{1}{N_{p}}\leq RP\leq1$, with higher values indicating a larger portion of the ROI consists of short runs (indicates a more fine texture). |
| $\text{GLV}=\sum_{i=1}^{N_{g}} \sum_{j=1}^{N_{r}} p(i,j\vert\theta)(i-\mu)^{2}$,  where $\mu=\sum_{i=1}^{N_{g}} \sum_{j=1}^{N_{r}} p(i,j\vert\theta)i$ | **GLV (Gray Level Variance)** measures the variance in gray level intensity for the runs. |
| $\text{RV}=\sum_{i=1}^{N_{g}} \sum_{j=1}^{N_{r}} p(i,j\vert\theta)(j-\mu)^{2}$,  where $\mu=\sum_{i=1}^{N_{g}} \sum_{j=1}^{N_{r}} p(i,j\vert\theta)j$ | **RV (Run Variance)** is a measure of the variance in runs for the run lengths. |
| $\text{RE}=-\sum_{i=1}^{N_{g}} \sum_{j=1}^{N_{r}} p(i,j\vert\theta)\log_{2}(p(i,j\vert\theta)+\epsilon)$ | **RE (Run Entropy)** measures the uncertainty/randomness in the distribution of run lengths and gray levels. A higher value indicates more heterogeneity in the texture patterns. |
| $\text{LGLRE}=\frac{\sum_{i=1}^{N_{g}} \sum_{j=1}^{N_{r}} \frac{\text{P}(i,j\vert\theta)}{i^{2}}}{N_{z}(\theta)}$ | **LGLRE (low gray level run emphasis)** measures the distribution of low gray-level values, with a higher value indicating a greater concentration of low gray-level values in the image. |
| $\text{HGLRE}=\frac{\sum_{i=1}^{N_{g}} \sum_{j=1}^{N_{r}} \text{P}(i,j\vert\theta)i^{2}}{N_{z}(\theta)}$ | **HGLRE (high gray level run emphasis)** measures the distribution of the higher gray-level values, with a higher value indicating a greater concentration of high gray-level values in the image. |
| $\text{SRLGLE}=\frac{\sum_{i=1}^{N_{g}} \sum_{j=1}^{N_{r}} \frac{\text{P}(i,j\vert\theta)}{i^{2}j^{2}}}{N_{z}(\theta)}$ | **SRLGLE (short run low gray level emphasis)** measures the joint distribution of shorter run lengths with lower gray-level values. |
| $\text{SRHGLE}=\frac{\sum_{i=1}^{N_{g}} \sum_{j=1}^{N_{r}} \frac{\text{P}(i,j\vert\theta)i^{2}}{j^{2}}}{N_{z}(\theta)}$ | **SRHGLE (short run high gray level emphasis)** measures the joint distribution of shorter run lengths with higher gray-level values. |
| $\text{LRLGLRE}=\frac{\sum_{i=1}^{N_{g}} \sum_{j=1}^{N_{r}} \frac{\text{P}(i,j\vert\theta)j^{2}}{i^{2}}}{N_{z}(\theta)}$ | **LRLGLRE (long run low gray level emphasis)** measures the joint distribution of long run lengths with lower gray-level values. |
| $\text{LRHGLRE}=\frac{\sum_{i=1}^{N_{g}} \sum_{j=1}^{N_{r}} \text{P}(i,j\vert\theta)i^{2}j^{2}}{N_{z}(\theta)}$ | **LRHGLRE (long run high gray level run emphasis)** measures the joint distribution of long run lengths with higher gray-level values. |
|  | |

***e. Gray Level Size Zone Matrix (GLSZM)***

A Gray Level Size Zone (GLSZM) describes gray level zones in a ROI, which are defined as the number of connected voxels that share the same gray level intensity. A voxel is considered connected if the distance is 1 according to the infinity norm (26-connected region in a 3D, 8-connected region in 2D). In a gray level size zone matrix ***P(i,j)*** the **(i,j)^th^** element equals the number of zones with gray level ***i*** and size ***j*** appear in image. Contrary to GLCM and GLRLM, the GLSZM is rotation independent, with only one matrix calculated for all directions in the ROI (Table S5).

Let:

**N_g_** be the number of discreet intensity values in the image

**N_s_** be the number of discreet zone sizes in the image

**N_p_** be the number of voxels in the image

**N_z_** be the number of zones in the ROI, which is equal to $\sum_{i=1}^{N_{g}} \sum_{j=1}^{N_{s}} \text{P}(i,j)$ and ***1 ≤ N_z_ ≤ N_p_***

***P(i,j)*** be the size zone matrix

***p(i,j)*** be the normalized size zone matrix, defined as $p(i,j)=\frac{\text{P}(i,j)}{N_{z}}$

***ϵ*** is an arbitrarily small positive number (≈2.2×10−16).

| **Table S5. Gray Level Size Zone Matrix (GLSZM)** | |
| --- | --- |
| **Radiomic feature** | **Interpretation** |
| $\text{SAE}=\frac{\sum_{i=1}^{N_{g}} \sum_{j=1}^{N_{s}} \frac{\text{P}(i,j)}{j^{2}}}{N_{z}}$ | **SAE (small area emphasis)** is a measure of the distribution of small size zones, with a greater value indicative of smaller size zones and more fine textures. |
| $\text{LAE}=\frac{\sum_{i=1}^{N_{g}} \sum_{j=1}^{N_{s}} \text{P}(i,j)j^{2}}{N_{z}}$ | **LAE (large area emphasis)** is a measure of the distribution of large area size zones, with a greater value indicative of larger size zones and more coarse textures. |
| $\text{GLN}=\frac{\sum_{i=1}^{N_{g}} {(\sum_{j=1}^{N_{s}} \text{P}(i,j))}^{2}}{N_{z}}$ | **GLN (gray level non-uniformity)** measures the variability of gray-level intensity values in the image, with a lower value indicating more homogeneity in intensity values. |
| $\text{GLNN}=\frac{\sum_{i=1}^{N_{g}} {(\sum_{j=1}^{N_{s}} \text{P}(i,j))}^{2}}{N_{z}^{2}}$ | **GLNN (gray level non-uniformity normalized)** measures the variability of gray-level intensity values in the image, with a lower value indicating a greater similarity in intensity values. This is the normalized version of the GLN formula. |
| $\text{SZN}=\frac{\sum_{j=1}^{N_{s}} {(\sum_{i=1}^{N_{g}} \text{P}(i,j))}^{2}}{N_{z}}$ | **SZN (size zone non-uniformity)** measures the variability of size zone volumes in the image, with a lower value indicating more homogeneity in size zone volumes. |
| $\text{SZNN}=\frac{\sum_{j=1}^{N_{s}} {(\sum_{i=1}^{N_{g}} \text{P}(i,j))}^{2}}{N_{z}^{2}}$ | **SZNN (size zone non-uniformity normalized)** measures the variability of size zone volumes throughout the image, with a lower value indicating more homogeneity among zone size volumes in the image. This is the normalized version of the SZN formula. |
| $\text{Zone Percentage}=\frac{N_{z}}{N_{p}}$ | **ZP (Zone Percentage)** measures the coarseness of the texture by taking the ratio of number of zones and number of voxels in the ROI. Values are in range $\frac{1}{N_{p}}\leq ZP\leq1$, with higher values indicating a larger portion of the ROI consists of small zones (indicates a more fine texture). |
| $\text{GLV}=\sum_{i=1}^{N_{g}} \sum_{j=1}^{N_{s}} p(i,j)(i-\mu)^{2}$, where $\mu=\sum_{i=1}^{N_{g}} \sum_{j=1}^{N_{s}} p(i,j)i$ | **Gray level variance (GLV)** measures the variance in gray level intensities for the zones. |
| $\text{ZV}=\sum_{i=1}^{N_{g}} \sum_{j=1}^{N_{s}} p(i,j)(j-\mu)^{2}$, where $\mu=\sum_{i=1}^{N_{g}} \sum_{j=1}^{N_{s}} p(i,j)j$ | **Zone Variance (ZV)** measures the variance in zone size volumes for the zones. |
| $\text{ZE}=-\sum_{i=1}^{N_{g}} \sum_{j=1}^{N_{s}} p(i,j)\log_{2}(p(i,j)+\epsilon)$ | **Zone Entropy (ZE)** measures the uncertainty/randomness in the distribution of zone sizes and gray levels. A higher value indicates more heterogeneneity in the texture patterns. |
| $\text{LGLZE}=\frac{\sum_{i=1}^{N_{g}} \sum_{j=1}^{N_{s}} \frac{\text{P}(i,j)}{i^{2}}}{N_{z}}$ | **LGLZE (low gray level zone emphasis)** measures the distribution of lower gray-level size zones, with a higher value indicating a greater proportion of lower gray-level values and size zones in the image. |
| $\text{HGLZE}=\frac{\sum_{i=1}^{N_{g}} \sum_{j=1}^{N_{s}} \text{P}(i,j)i^{2}}{N_{z}}$ | **HGLZE (high gray level zone emphasis)** measures the distribution of the higher gray-level values, with a higher value indicating a greater proportion of higher gray-level values and size zones in the image. |
| $\text{SALGLE}=\frac{\sum_{i=1}^{N_{g}} \sum_{j=1}^{N_{s}} \frac{\text{P}(i,j)}{i^{2}j^{2}}}{N_{z}}$ | **SALGLE (small area low gray level emphasis)** measures the proportion in the image of the joint distribution of smaller size zones with lower gray-level values. |
| $\text{SAHGLE}=\frac{\sum_{i=1}^{N_{g}} \sum_{j=1}^{N_{s}} \frac{\text{P}(i,j)i^{2}}{j^{2}}}{N_{z}}$ | **SAHGLE (small area high gray level emphasis)** measures the proportion in the image of the joint distribution of smaller size zones with higher gray-level values. |
| $\text{LALGLE}=\frac{\sum_{i=1}^{N_{g}} \sum_{j=1}^{N_{s}} \frac{\text{P}(i,j)j^{2}}{i^{2}}}{N_{z}}$ | **LALGLE (low area low gray level emphasis)** measures the proportion in the image of the joint distribution of larger size zones with lower gray-level values. |
| $\text{LAHGLE}=\frac{\sum_{i=1}^{N_{g}} \sum_{j=1}^{N_{s}} \text{P}(i,j)i^{2}j^{2}}{N_{z}}$ | **LAHGLE (low area high gray level emphasis)** measures the proportion in the image of the joint distribution of larger size zones with higher gray-level values. |
|  | |

***f. Neigbouring Gray Tone Difference Matrix (NGTDM)***

A Neighbouring Gray Tone Difference Matrix quantifies the difference between a gray value and the average gray value of its neighbours within distance ***δ*** (Table S6). The sum of absolute differences for gray level ***i*** is stored in the matrix. Let $\text{X}_{\mathbf{gl}}$be a set of segmented voxels and $x_{gl}(j_{x},j_{y},j_{z})\in\text{X}_{gl}$ be the gray level of a voxel at postion $(j_{x},j_{y},j_{z})$, then the average gray level of the neigbourhood is:

$$\begin{matrix} & \overline{A}_{i}=\overline{A}(j_{x},j_{y},j_{z}) \\ & =\frac{1}{W}\sum_{k_{x}=-\delta}^{\delta} \sum_{k_{y}=-\delta}^{\delta} \sum_{k_{z}=-\delta}^{\delta} x_{gl}(j_{x}+k_{x},j_{y}+k_{y},j_{z}+k_{z}), \\ & \text{where }(k_{x},k_{y},k_{z})\neq(0,0,0)\text{ and }x_{gl}(j_{x}+k_{x},j_{y}+k_{y},j_{z}+k_{z})\in\text{X}_{gl} \end{matrix}$$

Here, ***W*** is the number of voxels in the neighbourhood that are also in ***X_gl_***.

Let:

***n_i_*** be the number of voxels in ***X_gl_*** with gray level ***i***

***Nv,p*** be the total number of voxels in ***X_gl_*** and equal to $\sum n_{i}$ (i.e. the number of voxels with a valid region; at least 1 neighbor). $N_{v,p}\leq N_{p}$, where *N_p_* is the total number of voxels in the ROI.

***p_i_*** be the gray level probability and equal to $n_{i}/N_{v}$

$s_{i}=\{\begin{matrix} \sum^{n_{i}} \left| i-\overline{A}_{i} \right| & \text{for} & n_{i}\neq0 \\ 0 & \text{for} & n_{i}=0 \end{matrix}$ be the sum of absolute differences for gray level i

***N_g_*** be the number of discreet gray levels

***N_g,p_*** be the number of gray levels where ***p_i_***≠0

| **Table S6. Neigbouring Gray Tone Difference Matrix (NGTDM)** | |
| --- | --- |
| **Radiomic feature** | **Interpretation** |
| $\boldsymbol{Coarseness}=\frac{1}{\sum_{i=1}^{N_{g}} p_{i}s_{i}}$ | **Coarseness** is a measure of average difference between the center voxel and its neighbourhood and is an indication of the spatial rate of change. A higher value indicates a lower spatial change rate and a locally more uniform texture. |
| $\boldsymbol{Contrast}=\left( \frac{1}{N_{g,p}\left( N_{g,p}-1 \right)}\sum_{i=1}^{N_{g}} \sum_{j=1}^{N_{g}} p_{i}p_{j}(i-j)^{2} \right)\left( \frac{1}{N_{v,p}}\sum_{i=1}^{N_{g}} s_{i} \right)\text{, }$  $\text{where }p_{i}\neq0,p_{j}\neq0$ | **Contrast** is a measure of the spatial intensity change, but is also dependent on the overall gray level dynamic range. Contrast is high when both the dynamic range and the spatial change rate are high, i.e. an image with a large range of gray levels, with large changes between voxels and their neighbourhood. |
| $\boldsymbol{Busyness}=\frac{\sum_{i=1}^{N_{g}} p_{i}s_{i}}{\sum_{i=1}^{N_{g}} \sum_{j=1}^{N_{g}} \left\vert ip_{i}-jp_{j} \right\vert}\text{, }$  $\text{where }p_{i}\neq0,p_{j}\neq0$ | A measure of the change from a pixel to its neighbour. A high value for busyness indicates a ‘busy’ image, with rapid changes of intensity between pixels and its neighbourhood. |
| $\boldsymbol{Complexity}=\frac{1}{N_{v,p}}\sum_{i=1}^{N_{g}} \begin{aligned} \sum_{j=1}^{N_{g}} \left\vert i-j \right\vert\frac{p_{i}s_{i}+p_{j}s_{j}}{p_{i}+p_{j}}\text{, } \\ \end{aligned}$  $\text{where }p_{i}\neq0,p_{j}\neq0$ | An image is considered complex when there are many primitive components in the image, i.e. the image is non-uniform and there are many rapid changes in gray level intensity. |
| $\boldsymbol{Strength}=\frac{\sum_{i=1}^{N_{g}} \sum_{j=1}^{N_{g}} (p_{i}+p_{j})(i-j)^{2}}{\sum_{i=1}^{N_{g}} s_{i}}\text{, }$  $\text{where }p_{i}\neq0,p_{j}\neq0$ | **Strength** is a measure of the primitives in an image. Its value is high when the primitives are easily defined and visible, i.e. an image with slow change in intensity but more large coarse differences in gray level intensities. |
|  | |

***g. Gray Level Dependence Matrix (GLDM)***

A Gray Level Dependence Matrix (GLDM) quantifies gray level dependencies in an image. A gray level dependency is defined as the number of connected voxels within distance ***δ*** that are dependent on the center voxel. A neighbouring voxel with gray level ***j*** is considered dependent on center voxel with gray level ***i*** if ***|i−j|≤α***. In a gray level dependence matrix ***P(i,j)*** the ***(i,j)^th^*** element describes the number of times a voxel with gray level ***i*** with ***j*** dependent voxels in its neighbourhood appears in image (Table S7).

***N_g_*** be the number of discreet intensity values in the image

***N_d_*** be the number of discreet dependency sizes in the image

***N_z_*** be the number of dependency zones in the image, which is equal to $\sum_{i=1}^{N_{g}} \sum_{j=1}^{N_{d}} \text{P}(i,j)$

***P(i,j)*** be the dependence matrix

***p(i,j)*** be the normalized dependence matrix, defined as $p(i,j)=\frac{\text{P}(i,j)}{N_{z}}$

| **Table S7. Gray Level Dependence Matrix (GLDM)** | |
| --- | --- |
| **Radiomic feature** | **Interpretation** |
| $\boldsymbol{SDE}=\frac{\sum_{i=1}^{N_{g}} \sum_{j=1}^{N_{d}} \frac{\text{P}(i,j)}{i^{2}}}{N_{z}}$ | **SDE (Small Dependence Emphasis)**: A measure of the distribution of small dependencies, with a greater value indicative of smaller dependence and less homogeneous textures. |
| $\boldsymbol{LDE}=\frac{\sum_{i=1}^{N_{g}} \sum_{j=1}^{N_{d}} \text{P}(i,j)j^{2}}{N_{z}}$ | **LDE (Large Dependence Emphasis)**: A measure of the distribution of large dependencies, with a greater value indicative of larger dependence and more homogeneous textures. |
| $\boldsymbol{GLN}=\frac{\sum_{i=1}^{N_{g}} {(\sum_{j=1}^{N_{d}} \text{P}(i,j))}^{2}}{\sum_{i=1}^{N_{g}} \sum_{j=1}^{N_{d}} {\text{P}(i,j)}}$ | **GLN (Gray Level Non-Uniformity)**: Measures the similarity of gray-level intensity values in the image, where a lower GLN value correlates with a greater similarity in intensity values. |
| $\boldsymbol{DN}=\frac{\sum_{j=1}^{N_{d}} {(\sum_{i=1}^{N_{g}} \text{P}(i,j))}^{2}}{N_{z}}$ | **DN (Dependence Non-Uniformity)**: Measures the similarity of dependence throughout the image, with a lower value indicating more homogeneity among dependencies in the image. |
| $\boldsymbol{DNN}=\frac{\sum_{j=1}^{N_{d}} {(\sum_{i=1}^{N_{g}} \text{P}(i,j))}^{2}}{N_{z}^{2}}$ | **DNN (Dependence Non-Uniformity Normalized)**: Measures the similarity of dependence throughout the image, with a lower value indicating more homogeneity among dependencies in the image. This is the normalized version of the DLN formula. |
| $\boldsymbol{GLV}=\sum_{i=1}^{N_{g}} \sum_{j=1}^{N_{d}} p(i,j)(i-\mu)^{2}\text{, }$  $\text{where}\text{ }\mu=\sum_{i=1}^{N_{g}} \sum_{j=1}^{N_{d}} ip(i,j)$ | **GLV (Gray Level Variance)**: Measures the variance in grey level in the image. |
| $\boldsymbol{DV}=\sum_{i=1}^{N_{g}} \sum_{j=1}^{N_{d}} p(i,j)(j-\mu)^{2}\text{, }$  $\text{where}\text{ }\mu=\sum_{i=1}^{N_{g}} \sum_{j=1}^{N_{d}} jp(i,j)$ | **DV (Dependence Variance)**: Measures the variance in dependence size in the image. |
| $\boldsymbol{DE}=-\sum_{i=1}^{N_{g}} \sum_{j=1}^{N_{d}} p(i,j)\log_{2}(p(i,j)+\epsilon)$ | **DE (Dependence Entropy)**: Measures the entropy in dependence size in the image. |
| $\boldsymbol{LGLE}=\frac{\sum_{i=1}^{N_{g}} \sum_{j=1}^{N_{d}} \frac{\text{P}(i,j)}{i^{2}}}{N_{z}}$ | **LGLE (Low Gray Level Emphasis)**: Measures the distribution of low gray-level values, with a higher value indicating a greater concentration of low gray-level values in the image. |
| $\boldsymbol{HGLE}=\frac{\sum_{i=1}^{N_{g}} \sum_{j=1}^{N_{d}} \text{P}(i,j)i^{2}}{N_{z}}$ | **HGLE (High Gray Level Emphasis)**: Measures the distribution of the higher gray-level values, with a higher value indicating a greater concentration of high gray-level values in the image. |
| $\boldsymbol{SDLGLE}=\frac{\sum_{i=1}^{N_{g}} \sum_{j=1}^{N_{d}} \frac{\text{P}(i,j)}{i^{2}j^{2}}}{N_{z}}$ | **SDLGLE (Small Dependence Low Gray Level Emphasis)**: Measures the joint distribution of small dependence with lower gray-level values. |
| $\boldsymbol{SDHGLE}=\frac{\sum_{i=1}^{N_{g}} \sum_{j=1}^{N_{d}} \frac{\text{P}(i,j)i^{2}}{j^{2}}}{N_{z}}$ | **SDHGLE (Small Dependence High Gray Level Emphasis)**: Measures the joint distribution of small dependence with higher gray-level values. |
| $\boldsymbol{LDLGLE}=\frac{\sum_{i=1}^{N_{g}} \sum_{j=1}^{N_{d}} \frac{\text{P}(i,j)j^{2}}{i^{2}}}{N_{z}}$ | **LDLGLE (Large Dependence Low Gray Level Emphasis)**: Measures the joint distribution of large dependence with lower gray-level values. |
| $\boldsymbol{LDHGLE}=\frac{\sum_{i=1}^{N_{g}} \sum_{j=1}^{N_{d}} \text{P}(i,j)i^{2}j^{2}}{N_{z}}$ | **LDHGLE (Large Dependence High Gray Level Emphasis)**: Measures the joint distribution of large dependence with higher gray-level values. |
|  | |
